# Synthesis of 4′-Substituted-2′-Deoxy-2′-α-Fluoro Nucleoside Analogs as Potential Antiviral Agents

**DOI:** 10.3390/molecules25061258

**Published:** 2020-03-11

**Authors:** Mahesh Kasthuri, Chengwei Li, Kiran Verma, Olivia Ollinger Russell, Lyndsey Dickson, Louise McCormick, Leda Bassit, Franck Amblard, Raymond F. Schinazi

**Affiliations:** Center for AIDS Research, Laboratory of Biochemical Pharmacology, Department of Pediatrics, Emory University School of Medicine, 1760 Haygood Drive, Atlanta, GA 30322, USA; mahesh.kasthuri@emory.edu (M.K.); cli35@emory.edu (C.L.); kverma2@emory.edu (K.V.); olivia.ollinger@emory.edu (O.O.R.); lyndsey.dickson@emory.edu (L.D.); alouisemccormick@gmail.com (L.M.); leda.bassit@emory.edu (L.B.); famblar@emory.edu (F.A.)

**Keywords:** nucleoside, virus, polymerase inhibitors, respiratory syncytial virus, Zika, Dengue, West Nile, Chikungunya

## Abstract

Nucleoside analogs are widely used for the treatment of viral diseases (Hepatitis B/C, herpes and human immunodeficiency virus, HIV) and various malignancies. ALS-8176, a prodrug of the 4′-chloromethyl-2′-deoxy-2′-fluoro nucleoside ALS-8112, was evaluated in hospitalized infants for the treatment of respiratory syncytial virus (RSV), but was abandoned for unclear reasons. Based on the structure of ALS-8112, a series of novel 4′-modified-2′-deoxy-2′-fluoro nucleosides were synthesized. Newly prepared compounds were evaluated against RSV, but also against a panel of RNA viruses, including Dengue, West Nile, Chikungunya, and Zika viruses. Unfortunately, none of the compounds showed marked antiviral activity against these viruses.

## 1. Introduction

**Figure 1:**
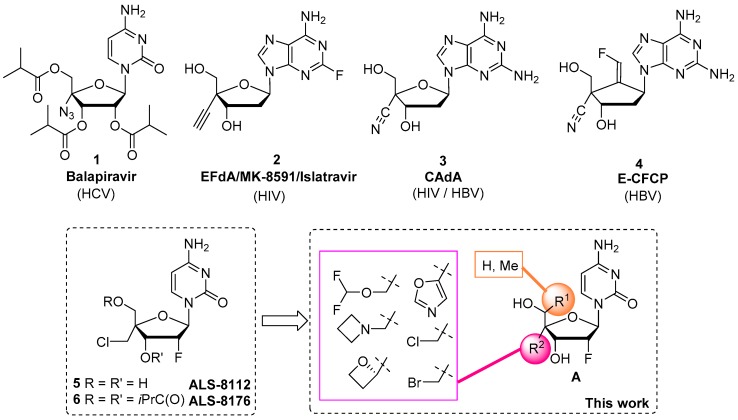
A selection of 4′-substituted nucleoside analogs displaying antiviral activity and structures of targeted 4′- and 5′-substituted-2′-deoxy-2′-fluoro cytidine analogs (A).

Modified nucleoside and nucleotide analogs are now the cornerstone of antiviral and anticancer chemotherapies [1,2] and among them, 4′-substituted nucleosides have attracted a great deal of attention (Figure 1). Balapiravir (1), the prodrug of 4′-azidocytidine, was one of the early hits identified as a potent and selective inhibitor of hepatitis C virus (HCV) RNA polymerase [3]. Further, 4′-ethynyl-2-fluoro-2′-deoxyadenosine (2) (EFdA/MK-8591/islatravir), in its triphosphate form, is a highly potent nucleoside reverse transcriptase translocation inhibitor (NRTTI) which is right now evaluated for the treatment and pre-exposure prophylaxis of HIV-1 infection via subdermal implant [4]. In addition, 4′-*C*-cyano-2-amino-2′-deoxyadenosine (CAdA) (3) [5] was also reported as a highly potent inhibitor of both HBV and HIV-1 replication while E-CFCP (4), another 4′-*C*-cyano nucleoside analog, was reported to be a subnanomolar inhibitor of HBV replication [6]. ALS-8176/lumicitabine (6), a prodrug of ALS-8112, a 4′-chloromethyl-2′-deoxy-2′-fluorocytidine analog, was evaluated in a phase 2 clinical trial for the treatment of respiratory syncytial virus (RSV) infections which was terminated for unclear reasons [7]. We recently reported that ALS-8112 also displayed potent anti-Nipah virus activity in vitro while also displaying in vitro toxicity [8]. Based on the potential of ALS-8112, we wish to report herein, the synthesis and the antiviral evaluation of new 4′-substituted-2′-deoxy-2′-fluoro cytidine nucleoside analogs. Although numerous 4′-substitutions have already been introduced on the ALS-8112 scaffold, these modifications remained basic and included mostly simple groups such as N_3_, alkyls, vinyl, ethynyl, cycloalkyl, ethers and thioethers. Through this work we focused on small groups that had never been introduced on the 4′-position of a nucleoside analog. These modifications included small heterocyclic rings (azetidine, oxetane and isoxazole), but also a unique difluoromethyl ether group. In parallel, we also evaluated the effect, in terms of antiviral potency, of a methyl group on the 5′-methylene portion of ALS-8112, a modification known to be tolerated by other viral polymerases [9].

## 2. Results

### 2.1. Chemistry

**Scheme 1:**
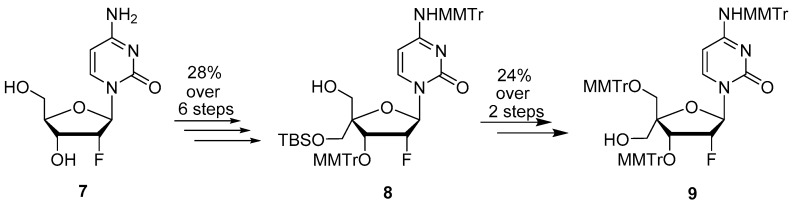
Synthesis of key intermediates **8** and **9** from commercially available 2′-deoxy-2′-α-fluorocytidine **7**.

**Scheme 2:**
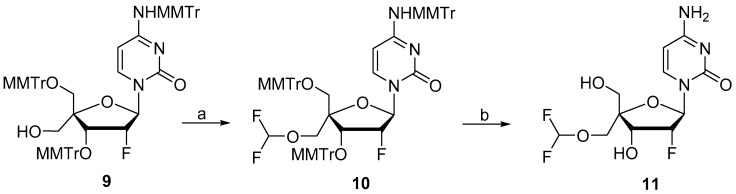
Synthesis of compound **11**. Reagents and conditions: (a) CuI, FSO_2_CF_2_CO_2_H, CH_3_CN, 60 °C, 2 h, 22%. (b) 80% aq AcOH, rt, 16 h, 68%.

**Scheme 3:**
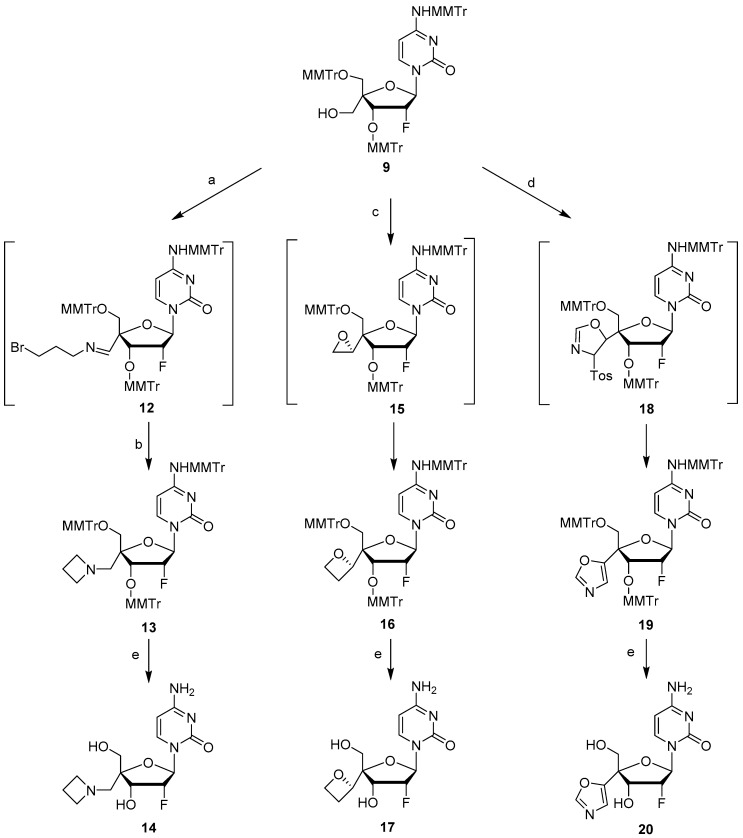
Synthesis of compounds **14**, **17** and **20**. Reagents and conditions: (a) (i) Dess-Martin periodinane, pyridine, DCM, 3 h, rt; (ii) 3-bromopropylamine, MgSO_4_, DCM, rt, 6 h, Quant. (b) NaBH_4_, 40 °C, 2 h, 72% over 3 steps; (c) (i) Dess-Martin periodinane, pyridine, DCM, 3 h, rt; (ii) trimethyloxosulfonium iodide, KO^t^Bu, ^t^BuOH, 65% over 2 steps. (d) (i) Dess-Martin periodinane, pyridine, DCM, 3 h, rt; (ii) tosylmethyl isocyanide, K_2_CO_3_, MeOH, reflux, 2 h, 74% over 2 steps. (e) 80% aq. AcOH, rt, 16 h, 14 (68%), 17 (73%) and 20 (76%).

**Figure 2:**
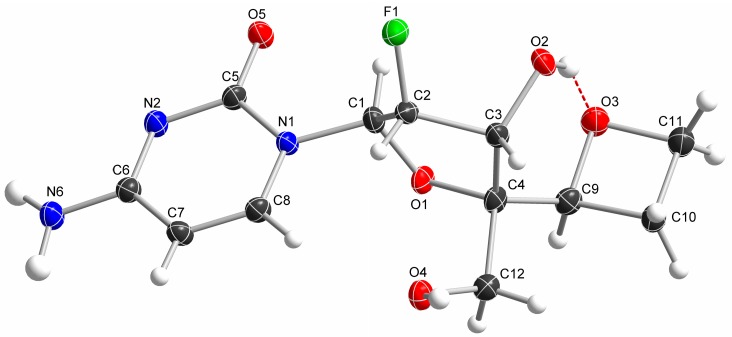
The ORTEP drawing of nucleoside 17 from X-ray crystal analysis.

Targeted 4′-substituted-2′-deoxy-2′-α-fluoro nucleoside derivatives 11, 14, 17, 20, 25, and 26 were prepared from key intermediates 8 and/or 9 obtained from commercially available 2′-deoxy-2′-α-fluorocytidine 7 following the chemistry described by Wang et al. [10] (Scheme 1). The synthesis of 4′-difluoromethoxy analog 11 was achieved by the reaction of 9 with a reactive Cu-difluorocarbene complex obtained by the reaction of CuI with FSO_2_CF_2_CO_2_H [11], followed by removal of the trityl groups in 80% aqueous acetic acid (Scheme 2). We first thought to prepare the desired azetidine analog 14 by reacting an activated 5′-methyltriflate intermediate with azetidine in presence of an organic base (Et_3_N or pyridine). However, under these conditions, we were unable to observe formation of the desired compound. We hypothesized that the relatively bulky azetidine ring could not reach the sterically hindered 5′- position due to the presence of the nearby large 5′- and 3′- monomethoxytrityl groups. Therefore, we subsequently evaluated an intramolecular reductive cyclization via the use of a primary halogeno alkylamine. The oxidation of 9 to the corresponding aldehyde with Dess Martin periodinane followed by reaction with 3-bromopropylamine in the presence of MgSO_4_ led to the formation of imine intermediate 12 which was subsequently reduced with NaBH_4_. Finally, the newly formed amine displaced the terminal bromine to form the desired azetidine derivative 13 [12,13]. Treatment of 13 under acidic conditions gave the targeted compound **14** (Scheme 3). The 4′-oxetane analog 17 was obtained from 9 by, first, oxidation to the corresponding aldehyde followed by a Johnson-Corey-Chaykovsky epoxidation and consecutive ring-expansion. Thus, compound **9** was oxidized by treatment with Dess-Martin periodinane to the corresponding aldehyde which was treated with 10 equivalents of trimethyloxosulfonium iodide in presence of *^t^*BuOK for 4 days to provide oxetane derivative 16 as a single isomer. Final deprotection under acidic conditions afforded the desired 4′-oxetane analog 17 in 48% yield over 3 steps (Scheme 3). Stereochemistry of the oxetane ring in compound **17** could not be assessed with certitude by NMR analysis, therefore, crystals were grown from methanol by slow evaporation. Results from X-ray structure determination of 17 led us to ascertain the *S*-configuration of the 5′-carbon (Figure 2). Synthesis of 4′-isoxazole analog 20 was achieved from intermediate 9 by first oxidation to the 5-aldehyde intermediate followed by a Van Leusen cyclization reaction using tosylmethyl isocyanide (TosMIC) in the presence of K_2_CO_3_ [14] and final deprotection with acetic acid.

**Scheme 4:**
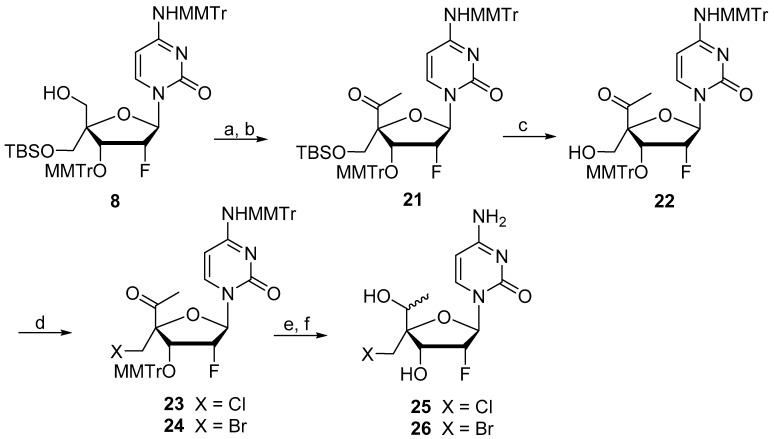
Synthesis of compounds **25** and **26**. Reagents and conditions: (a) (i) DCC, pyridine DMSO, TFA, rt, overnight; (ii) MeMgCl, THF, 86% over 2 steps. (b) DCC, pyridine DMSO, TFA, 87%. (c) TBAF, 95%; (d) (i) Tf_2_O, pyridine; (ii) LiX, DMF. (e) (i) NaBH_4_, MeOH; X = Cl 89%, X = Br 93%; (f) 80% aq. AcOH, 60 °C, 16 h, X = Cl 81%, X = Br 48%**.**

Targeted 5′-methyl derivatives 25 and 26 were prepared by following the chemistry described in Scheme 4. Protected intermediate 8 was oxidized under Pfitzner–Moffatt conditions and then reacted with MeMgCl to give the desired methylated intermediate as a 1/1 mixture. This compound was then oxidized to the corresponding ketone 21 under Pfitzner–Moffatt conditions and the *tert*-butyldimethylsilyl (TBS) group was removed using tetra-*n*-butylammonium fluoride (TBAF). **22** was then reacted with Tf_2_O in pyridine to form a triflate intermediate which was directly treated with LiCl or LiBr in DMF to give the corresponding halogeno derivatives 23 and 24, respectively. Finally, reduction with NaBH_4_ and removal of the monomethoxytrityl groups under acidic conditions afforded the desired compounds **25** and **26** as 1/1 mixtures of isomers at the 5′-position.

### 2.2. Antiviral and Toxicity Evaluation

Based on their structural similarity with ALS-8112, compounds **11**, **14**, **17**, **20**, **25** and **26** were tested against RSV replicon-containing adenocarcinomic human alveolar basal epithelial A549 cells (kindly provided by Apath, L.L.C, New York, NY, USA), but unfortunately none of them display antiviral activity in this system when evaluated up to 10 µM. It is worth noting that they did not show toxicity either, up to 100 µM, in a panel of cell lines, including primary human peripheral blood mononuclear (PBM) cells, human lymphoblastoid cells (CEM), African Green monkey (Vero) cells and human liver hepatocarcinoma (HepG2) cells. The excellent safety profile of these compounds led us to further evaluate them against a panel of RNA viruses (Dengue (DENV), West Nile (WNV), Chikungunya (CHIKV), and Zika viruses (ZIKV)) but, once again, none of them displayed antiviral activity when tested up to 20 µM for ZIKV or 30 µM for DENV, WNV, or CHIKV.

## 3. Experimental Section

### 3.1. Synthesis

Anhydrous solvents were purchased from Aldrich Chemical Company, Inc. (Milwaukee, Wisconsin, USA). Reagents were purchased from commercial sources. Unless noted otherwise, the materials used in the examples were obtained from readily available commercial suppliers or synthesized by standard methods known to one skilled in the art of chemical synthesis. ^1^H, ^13^C, and ^19^F spectra were taken on a Bruker Ascend^TM^ 400 spectrometer (Bruker BioSpin Corporation, Billerica, MA, USA) at rt and reported in ppm downfield from internal tetramethylsilane (for ^1^H-NMR). NMR processing was performed with MestReNova version 10.0.2-15465. Deuterium exchange and decoupling experiments were utilized to confirm proton assignments. Signal multiplicities are represented by s (singlet), d (doublet), dd (doublet of doublets), t (triplet), q (quadruplet), br (broad), bs (broad singlet), m (multiplet). All J-values are in Hz and calculated by Mnova or MestReNova programs (V 14.1.1). Mass spectra were determined on a Waters Acquity UPLC using electrospray ionization (Waters Corporation, Milford, MA, USA). Analytic TLC was performed on Analtech GHLF silica gel plates (Analtech, Newark, DE, USA), and preparative TLC on Analtech GF silica gel plates (Analtech, Newark, DE, USA). Column chromatography was performed on Combiflash R_f_200 or via reverse-phase high performance liquid chromatography. ^1^H, ^13^C and ^19^F-NMR spectra for compounds **11**, **14**, **17**, **20**, **25** and **26** are available online in Supplementary Materials at (Figures S1–S18).

*1-((2R,3R,4R,5S)-5-((Difluoromethoxy)methyl)-3-fluoro-4-((4-methoxyphenyl)diphenylmethoxy)-5-(((4-methoxyphenyl)diphenylmethoxy)methyl)tetrahydrofuran-2-yl)-4-(((4-methoxyphenyl) diphenylmethyl)amino)pyrimidin-2(1H)-one 10*: To a solution of **9** (650 mg, 0.59 mmol) in acetonitrile (10 mL) was added CuI (22.2 mg, 0.12 mol). The resulting reaction mixture was heated to 60 °C and a solution of 2,2-difluoro-2-(fluorosulfonyl) acetic acid (89.7 µL, 0.89 mmol) in acetonitrile (2 mL) was added slowly over 10 min. After 2 h at 60 °C, the reaction was cooled down to room temperature, quenched with a saturated solution of NaHCO_3_ (50 mL) and stirred for 30 min at this temperature. The solution was then extracted with ethyl acetate (3 × 20 mL) and the combined organic layers were washed with brine (20 mL), dried over Na_2_SO_4_, filtered and concentrated under reduced pressure. The resulting crude product was purified by flash column chromatography (dichloromethane/methanol, 100/0 to 95/5) to give compound **10** (150 mg, 22%). ^1^H NMR (400 MHz, CDCl_3_): δ 7.5–6.64 (m, 38H), 6.22 (t, *J* = 74.8, Hz, 1H), 6.14 (d, *J* = 7.6 Hz, 1H), 6.09 (d, *J* = 19.7 Hz, 1H), 4.54 (dd, *J* = 24.5 Hz, *J* = 5.0 Hz, 1H), 4.32 (q, *J* = 12.0 Hz, 2H), 4.24 (d, *J* = 7.6 Hz, 1H), 3.78 (s, 3H), 3.76 (s, 3H), 3.73 (s, 3H), 3.61 (dd, *J* = 70.6 Hz, *J* = 10.0 Hz, 1H), 3.22 (dd, *J* = 52.0 Hz, *J* = 5.0 Hz, 1H). ^19^F NMR (376 MHz, CDCl_3_): δ –84.11 (d, *J* = 74.8 Hz), −185.8 (m). ^13^C NMR (101 MHz, CDCl_3_): δ 165.3, 159.1, 158.7, 158.6, 154.4, 144.3, 144.0, 143.8, 143.67, 143.6, 143.3, 141.3, 135.8, 134.5, 134.3, 131.0, 130.8, 130.7, 129.9, 128.8, 128.7, 128.6, 128.5, 128.4, 128.3, 128.2, 127.9, 127.8, 127.6, 127.5, 127.4, 127.1, 127.0, 118.7, 116.1, 113.5, 113.4, 113.3, 113.2, 112.9, 94.8, 94.0, 89.4, 89.1, 88.2, 88.1, 87.4, 86.7, 72.2, 72.0, 703, 64.3, 62.3, 55.2. HRMS for C_71_H_62_F_3_N_3_O_8_ (M + H]. Calcd: *m*/*z* 1142.4489, found: *m*/*z* 1142.4549.

*4-Amino-1-((2R,3R,4R,5S)-5-((difluoromethoxy)methyl)-3-fluoro-4-hydroxy-5-(hydroxymethyl) tetrahydrofuran-2-yl)pyrimidin-2(1H)-one 11*: Compound **10** (150 mg, 0.13 mmol) was treated with 3 mL of 80% acetic acid in water (*v*/*v*) at 50–60 °C for 12 h. Volatiles were evaporated under reduced pressure and the residue co-evaporated with toluene (3 × 5 mL). The resulting crude product was purified by flash column chromatography (dichloromethane/methanol, 100/0 to 90/10) to give compound **11** (29 mg, 68%). ^1^H NMR (400 MHz, MeOD-*d_4_*): δ 8.09 (d, *J* = 7.6 Hz, 1H), 6.43 (t, *J* = 75.6, Hz, 1H), 6.17 (dd, *J* = 15.4 Hz, *J* = 3.6 Hz, 1H), 5.93 (d, *J* = 7.5 Hz, 1H), 5.17 (dq, *J* = 53.5 Hz, *J* = 3.6 Hz, 1H), 4.56 (dd, *J* = 15.5 Hz, *J* = 5 Hz, 1H), 4.09 (q, *J* = 12.0 Hz, 2H), 4.09 (dd, *J* = 24.0 Hz, *J* = 11.2 Hz, 2H). ^19^F NMR (376 MHz, MeOD-*d*_4_): δ −85.6 (d, *J* = 73.0 Hz), -186.68 (m). ^13^C NMR (101 MHz, MeOD-*d*_4_): δ165.2, 156.8, 142.2, 120.2, 117.7, 115.2, 94.9, 94.5, 92.7, 87.4, 87.1, 86.5, 69.3, 69.2, 65.2, 61.5. HRMS for C_11_H_14_F_3_N_3_O_5_ (M + H]. Calcd: *m*/*z* 326.0885, found: *m*/*z* 326.0950.

*1-((2R,3R,4R,5R)-5-(Azetidin-1-ylmethyl)-3-fluoro-4-((4-methoxyphenyl)diphenylmethoxy)-5-(((4-methoxyphenyl)diphenylmethoxy)methyl)tetrahydrofuran-2-yl)-4-(((4-methoxyphenyl) diphenylmethyl)amino)pyrimidin-2(1H)-one 13*: To a solution of 9 (200 mg, 0.18 mmol) in dichloromethane (7 mL) was added pyridine (0.14 mL, 1.83 mmol) and Dess-Martin periodinane (173 mg, 0.41 mmol) at 0 °C. The resulting reaction mixture was stirred at room temperature for 3 h, then quenched with 10 mL of Na_2_S_2_O_3_ and Na_2_CO_3_ (1:1 mixture). The solution was extracted with dichloromethane (3 × 20 mL) and the combined organic layers were washed with brine (20 mL), dried over Na_2_SO_4_, filtered and concentrated under reduced pressure. The resulting crude product was purified by flash column chromatography (dichloromethane/methanol, 100/0 to 95/5) to give the desired aldehyde (168 mg, 84%). To a solution of the freshly prepared aldehyde (168 mg, 0.15 mmol) in dichloromethane (5 mL) was added MgSO_4_ (170 mg). After 5 min, 3-bromopropylamine hydrogen chloride (37 mg, 0.17 mmol) and pyridine (19 µL, 0.23 mmol) were added and the resulting reaction mixture was stirred at room temperature for 16 h. After completion, the reaction was filtered through celite and concentrated under reduced pressure. The resulting crude product was dissolved in methanol (5 mL) before addition of sodium borohydride (6 mg, 0.15 mmol). The reaction mixture was then stirred for 2 h at 40 °C before being quenched at room temperature with a saturated solution of ammonium chloride (7 mL). The mixture was then diluted with ethyl acetate (35 mL). The organic layer was separated, washed with brine (7 mL), dried over Na_2_SO_4_, filtered and concentrated under reduced pressure. The resulting crude product was purified by flash column chromatography (dichloromethane/methanol, 100/0 to 95/5) to give compound **13** (149 mg, 86 %). ^1^H NMR (400 MHz, CDCl_3_): δ 7.44–6.60 (m, 38H), 6.20 (d, *J* = 18.0 Hz, 1H), 4.45 (dd, *J* = 27.0 Hz, *J* = 3.8 Hz, 1H), 4.08 (d, *J* = 18.0 Hz, 1H), 3.9 (d, *J* = 10.0 Hz, 1H), 3.77 (s, 6H), 3.73 (s, 3H), 3.59 (d, *J* = 10.0 Hz, 1H), 3.12–2.96 (m, 6H), 2.86 (d, *J* = 13.2 Hz, 1H), 1.91 (m, 2H). ^19^F NMR (376 MHz, CDCl_3_): δ −185.36 (m). ^13^C NMR (101 MHz, CDCl_3_): δ 165.1, 159.1,158.6, 158.5, 154.7, 144.4, 144.3, 144.0, 143.9, 143.7, 141.1,135.7, 134.8, 134.3, 131.0, 130.9, 129.9, 128.9, 128.8, 128.7, 128.6, 128.5, 128.4, 128.2, 128.1, 127.8, 127.7, 127.6, 127.5, 127.4, 127.3, 126.9, 126.8, 113.3, 113.1, 112.8, 94.7, 94.6, 92.7, 88.7, 87.8, 87.5, 71.5, 71.4, 70.1, 62.9, 60.6, 55.2, 18.8. HRMS for C_73_H_67_FN_4_O_7_ (M + H]. Calcd: *m*/*z* 1131.4994, found: *m*/*z* 1131.5055.

*4-Amino-1-((2R,3R,4R,5R)-5-(azetidin-1-ylmethyl)-3-fluoro-4-hydroxy-5-(hydroxymethyl) tetrahydrofuran-2-yl)pyrimidin-2(1H)-one 14*: Compound **13** (230 mg, 0.2 mmol) was treated with 4 mL of 80% acetic acid in water (*v*/*v*) at 50–60 °C for 12 h. Volatiles were evaporated under reduced pressure and co-evaporated with toluene (3 × 5 mL). The resulting crude product was purified by flash column chromatography (dichloromethane/methanol, 100/0 to 90/10) to afford 14 (43 mg, 68%). ^1^H NMR (400 MHz, MeOD-*d*_4_): δ 8.02 (d, *J* = 7.5 Hz, 1H), 6.18 (dd, *J* = 15.2 Hz, *J* = 3.6 Hz, 1H), 5.9 (d, *J* = 7.5 Hz, 1H), 5.1 (dq, *J* = 53.6 Hz, *J* = 3.7 Hz, 1H), 4.45 (dd, *J* = 15.7 Hz, *J* = 5.2 Hz, 1H), 3.71 (dd, *J* = 81.0 Hz, *J* = 11.8 Hz, 2H), 3.38 (m, 4H), 2.88 (dd, *J* = 14.5 Hz, *J* = 13.6 Hz, 2H), 2.14 (m, 2H). ^19^F NMR (376 MHz, MeOD-*d*_4_): δ −204.24 (dt, *J* = 53.6 Hz, *J* = 15.4 Hz). ^13^C NMR (101 MHz, MeOD-*d*_4_): δ166.4, 156.8, 141.8, 94.8, 94.7, 92.9, 88.5, 88.1, 86.8, 71.0, 70.9, 63.7, 60.7, 60.6, 56.5, 17.8. HRMS for C_13_H_19_FN_4_O_4_ (M + H]. Calcd: *m*/*z* 315.1390, found: *m*/*z* 315.1456.

*(2R,3R,4R,5S)-3-Fluoro-4-((4-methoxyphenyl)diphenylmethoxy)-5-(((4-methoxyphenyl) diphenylmethoxy)methyl)-5-((S)-oxetan-2-yl)tetrahydrofuran-2-yl)-4-(((4-methoxyphenyl) diphenylmethyl)amino)pyrimidin-2(1H)-one 16*: To a solution of 9 (200 mg, 0.18 mmol) in dichloromethane (7 mL) was added pyridine (0.14 mL, 1.83 mmol) and Dess-Martin periodinane (173 mg, 0.41 mmol) at 0 °C. The resulting reaction mixture was stirred at room temperature for 3 h, then quenched with 10 mL of Na_2_S_2_O_3_ and Na_2_CO_3_ (1:1 mixture). The solution was extracted with dichloromethane (3 × 20 mL) and the combined organic layers were washed with brine (20 mL), dried over Na_2_SO_4_, filtered and concentrated under reduced pressure. The resulting crude product was purified by flash column chromatography (dichloromethane/methanol, 100/0 to 95/5) to give the desired aldehyde intermediate (168 mg, 84%). A solution of trimethyl oxosulfonium iodide (0.425 g, 1.91 mmol) and potassium *tert*-butoxide (0.43 g, 3.83 mmol) in 3 mL of *tert*-butanol was stirred at 30 °C for 30 min before addition of the freshly prepare aldehyde (168 mg, 0.19 mmol) in *tert*-butanol (3 mL). The resulting mixture was stirred at 50 °C for 4 days. After being cooled down to room temperature, the mixture was poured into a saturated solution of ammonium chloride (10 mL) and then extracted with dichloromethane (2 × 10 mL). The combined organic layers were dried over sodium sulfate, filtered, and concentrated under reduced pressure. The resulting crude product was purified by flash column chromatography (dichloromethane/methanol, 100/0 to 97/3) to give 16 (133 mg, 78%). ^1^H NMR (400 MHz, CDCl_3_): δ 6.4–7.5 (m, 44H), 5.4 (t, *J* = 7.6 Hz, 1H), 4.62 (m, 2H), 4.40 (m, 1H), 3.95 (m, 2H), 3.80 (s, 3H), 3.77 (s, 3H), 3.74 (s, 3H), 3.63 (d, *J* = 10.6 Hz, 1H), 2.84 (m, 1H), 2.68 (m, 1H). ^19^F NMR (376 MHz, CDCl_3_): δ −186.83 (dt, *J* = 53.4, 25.6 Hz). ^13^C NMR(101 MHz, CDCl_3_): δ 165.0, 159.1, 158.7, 158.5, 154.7, 144.3, 144.2, 143.8, 143.6, 143.3, 143.2, 141.1, 135.6, 134.7, 134.2, 130.9, 130.8, 129.9, 129.2, 129.0, 128.8, 128.7, 128.4, 128.3, 128.2, 128.2, 127.9, 127.9, 127.7, 127.6, 127.4, 127.3, 127.2, 127.15, 113.4, 113.2, 112.7, 95.0, 94.2, 92.3, 89.6, 88.0, 87.8, 87.6, 87.4, 82.7, 77.2, 71.3, 71.2, 70.1, 69.1, 62.0, 55.2, 42.7, 42.7, 24.5. HRMS for C_72_H_65_FN_3_O_8_ (M + H). Calcd: *m*/*z* 1118.4756, found: *m*/*z* 1118.4758.

*4-Amino-1-((2R,3R,4R,5R)-3-fluoro-4-hydroxy-5-(hydroxymethyl)-5-((S)-oxetan-2-yl) tetrahydrofuran-2-yl)pyrimidin-2(1H)-one 17*. Compound **16** (610 mg, 0.546 mmol) was treated with 10 mL of 80% acetic acid in water (*v*/*v*) at 50–60 °C for 12 h. The volatiles were then evaporated under reduced pressure and the residue was purified by flash column chromatography (dichloromethane/methanol, 100/0 to 85/15) to give compound **17** (119 mg, 73%). ^1^H NMR (400 MHz, DMSO-d_6_): δ 7.89 (d, *J* = 7.4 Hz, 1H), 7.32 (NH_2_, 2H), 6.35 (dd, *J* = 11.4, 7.0 Hz, 1H), 5.80 (d, *J* = 7.4 Hz, 1H), 5.56 (br s, 1H), 5.25 (br s, 1H), 5.12 (ddd, *J* = 53.2, 6.8, 1.6 Hz, 1H), 4.96 (t, *J* = 7.4 Hz, 1H), 3.54 (m, 1H), 3.45 (m, 1H), 2.74 (m, 1H), 2.54 (m, 1H). ^19^F NMR (376 MHz, DMSO-d_6_): δ −210.35, (ddd, *J* = 53.2, 12, 3.6 Hz). ^13^C NMR (101 MHz, DMSO-d_6_): δ 166.1, 155.8, 141.8, 95.4, 93.1, 91.2, 89.29, 89.26, 86.3, 86.0, 82.6, 70.6, 70.4, 69.0, 61.8, 24.9. HRMS for C_12_H_17_FN_3_O_5_ (M + H). Calcd: *m*/*z* 302.1152, found: *m*/*z* 302.1142.

*1-((2R,3R,4R,5R)-3-Fluoro-4-((4-methoxyphenyl)diphenylmethoxy)-5-(((4-methoxyphenyl) diphenylmethoxy)methyl)-5-(oxazol-5-yl)tetrahydrofuran-2-yl)-4-(((4-methoxyphenyl) diphenylmethyl)amino)pyrimidin-2(1H)-one 19*: To a solution of 9 (200 mg, 0.18 mmol) in dichloromethane (7 mL) was added pyridine (0.14 mL, 1.83 mmol) and Dess-Martin periodinane (173 mg, 0.41 mmol) at 0 °C. The resulting reaction mixture was stirred at room temperature for 3 h, then quenched with 10 mL of Na_2_S_2_O_3_ and Na_2_CO_3_ (1:1 mixture). The solution was extracted with dichloromethane (3 × 20 mL) and the combined organic layers were washed with brine (20 mL), dried over Na_2_SO_4_, filtered and concentrated under reduced pressure. The resulting crude product was purified by flash column chromatography (dichloromethane/methanol, 100/0 to 95/5) to give the desired aldehyde intermediate (168 mg, 84%). To a solution of this aldehyde (168 mg, 0.15 mmol) in methanol (2 mL) was added sequentially *p*-toluenesulfonylmethyl isocyanide (TosMIC) (30 mg, 0.15 mmol) and K_2_CO_3_ (63 mg, 0.45 mmol). After 2 h at 65 °C, the volatiles were evaporated under reduced pressure, water (5 mL) was added and the solution stirred for 5 min. The organic content was extracted with ethyl acetate (3 × 5 mL), combined organic layer were washed with brine (5 mL), dried over Na_2_SO_4_, filtered and concentrated under reduced pressure. The resulting crude product was purified by flash column chromatography (dichloromethane/methanol, 100/0 to 95/5) to give 19 (147 mg, 88%). ^1^H NMR (400 MHz, CDCl_3_): δ 7.93 (s, 1H), 7.73–6.9 (m, 39H), 6.77 (d, *J* = 12 Hz, 2H), 6.70 (d, *J* = 12 Hz, 4H), 6.05 (d, *J* = 20.4 Hz, 1H), 4.66 (dd, *J* = 21.6 Hz, *J* = 4.4 Hz, 1H), 4.47 (d, *J* = 7.6 Hz, 1H), 3.77 (s, 3H), 3.76 (s, 3H), 3.74 (s, 3H), 3.68 (q, *J* = 10.0 Hz, 2H), 3.41 (dd, *J* = 51.6 Hz, *J* = 24.8 Hz, 1H). ^19^F NMR (376 MHz, CDCl_3_): δ −185.53 (m). ^13^C NMR (101 MHz, CDCl_3_): δ 165.5, 159.0, 158.7, 158.6, 154.3, 150.7, 150.2, 144.2, 144.1, 143.9, 143.8, 143.7, 143.2, 141.9, 135.7, 134.6, 134.4, 130.9, 130.6, 129.9, 128.8, 128.7, 128.6, 128.5, 128.4, 128.3, 128.2, 127.9, 127.8, 127.6, 127.5, 127.3, 127.1, 127.0, 125.3, 113.5, 113.1, 112.9, 94.9, 93.4, 91.7, 91.5, 91.4, 88.1, 87.1, 85.5, 73.6, 73.5, 70.4, 64.6, 55.2, 55.1. HRMS for C_72_H_61_FN_4_O_8_ (M + H]. Calcd: *m*/*z* 1129.45, found: *m*/*z* 1129.4543.

*4-Amino-1-((2R,3R,4R,5R)-3-fluoro-4-hydroxy-5-(hydroxymethyl)-5-(oxazol-5-yl)tetrahydrofuran-2-yl)pyrimidin-2(1H)-one 20*: Compound **19** (400 mg, 0.35 mmol) was treated with 8 mL of 80% acetic acid in water (**v*/*v**) at 50–60 °C for 12 h. The volatiles were then evaporated under reduced pressure and the residue co-evaporated with toluene (3 × 10 mL). The resulting crude product was purified by flash column chromatography (dichloromethane/methanol, 100/0 to 90/10) to give compound **20** (83.6 mg, 76%). ^1^H NMR (400 MHz, MeOD-*d_4_*): δ 8.21 (s, 1H), 8.05 (d, *J* = 7.5 Hz, 1H), 7.22 (s, 1H), 6.23 (dd, *J* = 18.0 Hz, *J* = 2.0 Hz, 1H), 5.93 (d, *J* = 7.5 Hz, 1H), 5.17 (dq, *J* = 53.5 Hz, *J* = 2.0 Hz, 1H), 4.79 (dd, *J* = 20.0 Hz, *J* = 5 Hz, 1H), 3.98 (q, *J* = 12.0 Hz, 2H). ^19^F NMR (376 MHz, MeOD-*d_4_*): δ −198.45 (dt, *J* = 53.0 Hz, *J* = 19.6 Hz). ^13^C NMR (101 MHz, MeOD-*d_4_*): δ 166.5, 156.5, 151.8, 150.0, 142.3, 124.1, 94.9, 94.6, 92.7, 90.8, 90.5, 85.9, 70.4, 70.3, 63. 3. HRMS for C_12_H_13_FN_4_O_5_ (M + H]. Calcd: *m*/*z* 313.09, found: *m*/*z* 313.0935.

*1-((2R,3R,4R,5S)-5-Acetyl-5-(((tert-butyldimethylsilyl)oxy)methyl)-3-fluoro-4-((4-methoxyphenyl) diphenylmethoxy)tetrahydrofuran-2-yl)-4-(((4-methoxyphenyl)diphenylmethyl)amino) pyrimidin-2(1H)-one 21*: To a solution of pyridine (0.21 mL, 2.51 mmol) in DMSO (4 mL) was added TFA (0.16 mL, 2.12 mmol) at 0 °C. The mixture was then stirred at room temperature for 10 min before being added dropwise to a solution of 8 (1.8 g, 1.93 mmol) and DCC (1.473 g, 7.14 mmol) in DMSO (10 mL). The reaction mixture was stirred overnight, quenched by adding water (20 mL) and ethyl acetate (20 mL). The precipitate was removed by filtration and washed with ethyl acetate (30 mL). The filtrate was extracted with dichloromethane (3 × 100 mL) and the combined organic layers were washed with a saturated solution of NaHCO_3_ (50 mL), dried with Na_2_SO_4_. filtered and concentrated under reduced pressure. The resulting residue was purified by flash column chromatography (dichloromethane/methanol, 100/0 to 95/5) to give the desired crude aldehyde. To a solution of this aldehyde (1.565 g, 1.68 mmol) in THF (10 mL) at −78 °C was slowly added MeMgCl (3.0 M solution in THF, 5.6 mL, 16.8 mmol). The mixture was stirred for 30 min at room temperature and then quenched at −78 °C with methanol (5 mL). The volatiles were then evaporated under reduced pressure and the residue was purified by flash column chromatography (dichloromethane/methanol, 100/0 to 98/2) to give the desired hydroxy intermediate as a 1:1 mixture of isomers (1.44 g, 91%). To a solution of pyridine (0.282 mL, 3.5 mmol) in DMSO (3 mL) was added TFA (0.227 mL, 2.96 mmol) at 0 °C. The mixture was then stirred at room temperature for 10 min before being added dropwise to a solution of the freshly prepared hydroxy intermediate (1.44 g, 1.52 mmol) and DCC (3.12 g, 5.62 mmol) in DMSO (10 mL). The reaction mixture was stirred overnight and quenched by adding water (15 mL) and ethyl acetate (15 mL). The precipitate was removed by filtration and washed with ethyl acetate (15 mL). The filtrate was extracted with dichloromethane (3 × 50 mL) and the combined organic layers were washed with a saturated solution of NaHCO_3_ (30 mL), dried with Na_2_SO_4_. filtered and concentrated under reduced pressure. The resulting residue was purified by flash column chromatography (dichloromethane/methanol, 100/0 to 95/5) to give 21 (1.25 g, 87%). ^1^H NMR (400 MHz, CDCl_3_) δ: 6.6–7.2 (m, 29H), 5.22 (d, *J* = 21.88 Hz, 1H), 4.87 (d, *J* = 7.52 Hz, 1H), 4.52 (dd, *J* = 22.32, 5.0 Hz, 1H), 4.37 (d, *J* = 11.4 Hz, 1H), 4.09 (d, *J* = 11.4 Hz, 1H), 4.05 (dd, *J* =53.64, 5.0 Hz, 1H), 3.69 (s, 1H), 3.08 (s, 3H), 1.89 (s, 3H), 0.78 (s, 9H), −0.0001 (s, 3H), -0.0222 (s, 3H). ^19^F NMR (376 MHz, CDCl_3_): δ −182.4 (dt, *J* = 53.6, 22.3Hz). ^13^C NMR (101 MHz CDCl_3_): δ 207.3, 165.9, 158.9, 158.8, 154.1, 144.2, 144.09, 144.05, 143.99, 143.6, 135.7, 134.8, 131.1, 130.0, 129.1, 128.9, 128.6, 128.4, 127.8, 127.7, 127.6, 127.4, 127.4, 127.3, 113.7, 113.1, 95.8, 95.4, 94.6, 92.7, 92.4, 90.5, 88.4, 73.5, 73.3, 70.6, 63.12, 63.09, 55.3, 49.2, 34.0, 26.2, 26.0, 25.6, 25.0, 18.4, −5.3, −5.4. HRMS for C_57_H_61_FN_3_O_7_Si (M + H). Calcd: *m*/*z* 946.4263, found: *m*/*z* 946.4250.

*1-((2R,3R,4R,5S)-5-Acetyl-3-fluoro-5-(hydroxymethyl)-4-((4-methoxyphenyl)diphenylmethoxy) tetrahydrofuran-2-yl)-4-(((4-methoxyphenyl)diphenylmethyl)amino)pyrimidin-2(1H)-one 22*: To a solution of 21 (1.0 g, 1.06 mmol) in THF (5 mL) was added TBAF (1M in THF, 2 mL, 2.0 mmol) at 0 °C. The mixture was stirred at room temperature for 7 h, water (30 mL) was added and extracted with dichloromethane (3 × 30 mL). The combined organic layers were dried over sodium sulfate, filtered and concentrated under reduced pressure. The resulting crude product was purified by flash column chromatography (dichloromethane/methanol, 100/0 to 98/2) to give compound **22** (837 mg, 95%). ^1^H NMR (400 MHz, CDCl_3_): δ 6.8–7.26 (m, 29H), 5.24 (d, *J* = 23.8 Hz, 1H), 5.04 (dd, *J* = 20.0, 5.5 Hz, 1H), 5.0 (d, *J* = 7.5 Hz, 1H), 4.1-4.4 (m, 3H), 3.79 (s, 6H), 2.03 (s, 3H), ^19^F NMR (376 MHz, CDCl_3_): δ −180.5, (dt, *J* = 56.9, 26.4 Hz), ^13^C NMR (101 MHz CDCl_3_): δ 208.5, 166.1, 159.0, 158.9, 154.0, 145.0, 144.00, 143.96, 143.93, 143.50, 135.6, 134.6, 131.1, 130.0, 128.8, 128.7, 128.5, 128.4, 128.0, 127.9, 127.7, 127.5, 127.4, 113.7, 113.2. 97.3, 97.0, 94.8, 92.4, 90.5, 88.5, 73.8, 73.6, 70.7, 62.4, 55.30, 55.28, 25.1. HRMS for C_51_H_47_FN_3_O_7_ (M + H). Calcd: *m*/*z* 832.3398, found: *m*/*z* 832.3388.

*1-((2R,3R,4R,5S)-5-Acetyl-5-(chloromethyl)-3-fluoro-4-((4-methoxyphenyl)diphenylmethoxy) tetrahydrofuran-2-yl)-4-(((4-methoxyphenyl)diphenylmethyl)amino)pyrimidin-2(1H)-one 23*: 22 (128 mg, 0.154 mmol) was co-evaporated with toluene twice then dissolved in dichloromethane (3 mL). Pyridine (0.14 mL, 1.54 mmol) was added to the solution and the mixture was cooled to −78 °C. Triflic anhydride (52 µL, 0.3 mmol) was then added and the mixture was stirred at 0 °C for 40 min. The volatiles were then evaporated under reduced pressure and the residue was dissolved in DMF (3 mL) before addition of LiCl (33 mg, 0.77 mmol). The mixture was stirred overnight, quenched with a saturated solution of NaHCO_3_ (20 mL) and extracted with dichloromethane (3 × 20 mL). The combined organic layers were dried over sodium sulfate, filtered and concentrated under reduced pressure. The resulting crude product was purified by flash column chromatography (Hexanes/ Ethyl acetate, 100/0 to 50/50) to give compound **23** (113 mg, 87%). ^1^H NMR (400 MHz, CDCl_3_): δ 6.8–7.3 (m, 29H), 5.14 (dd, *J* = 20.8, 5.0 Hz, 1H), 5.07 (d, *J* = 24.2 Hz, 1H), 5.01 (1H, d, *J* = 7.5 Hz, 1H), 4.65 (d, *J* = 12.1 Hz, 1H), 4.27 (dd, *J* = 53.9, 5.0 Hz, 1H), 3.98 (d, *J* = 12.1 Hz, 1H), 3.80 (s, 6H), 3.48 (s, 1H), 1.99 (s, 3H), ^19^F NMR (376 MHz, CDCl_3_): δ 179.8 (ddd, *J* = 57.8, 26, 22.9 Hz). ^13^C NMR (101 MHz CDCl_3_): δ 205.0, 166.1, 159.0, 158.9, 153.9, 145.2, 143.9, 143.8, 143.4, 135.6, 134.4, 131.3, 129.9, 128.8, 128.51, 128.47, 127.9, 127.8, 127.7, 127.41, 127.35, 113.7, 113.2, 97.1, 96.8, 94.9, 92.5, 91.3, 90.6, 88.6, 74.5, 74.4, 70.7, 55.29, 55.27, 50.9, 43.3, 43.2, 24.9. HRMS for C_51_H_46_ClFN_3_O_6_ (M + H). Calcd: *m*/*z* 850.3059, found: *m*/*z* 850.3052, 852.3044.

*1-((2R,3R,4R,5S)-5-Acetyl-5-(bromomethyl)-3-fluoro-4-((4-methoxyphenyl)diphenylmethoxy) tetrahydrofuran-2-yl)-4-(((4-methoxyphenyl)diphenylmethyl)amino)pyrimidin-2(1H)-one 24*: Title compound **24** was obtained from 22 using the same procedure as for compound **23** and replacing LiCl by LiBr. Yield: 88%. ^1^H NMR (400 MHz, CDCl_3_): δ 6.8–7.3 (m, 29H), 5.15 (dd, *J* = 20.9, 5.0 Hz, 1H), 5.06 (d, *J* = 25.5, 1H), 5.01 (d, *J* = 7.6 Hz, 1H), 4.49 (d, *J* = 11.3 Hz, 1H), 4.29 (dd, *J* = 55.6, 11.3 Hz, 1H), 3.85 (d, *J* = 11.3 Hz, 1H), 3.80 (s, 6H), 1.97 (s, 3H). ^19^F NMR (376 MHz, CDCl_3_): δ 205.0 ^13^C NMR (101 MHz CDCl_3_): δ 205.0, 166.1, 159.0, 158.9, 145.2, 143.9, 143.8, 143.4, 135.5, 134.4, 131.3, 129.9, 129.0, 128.8, 128.5, 128.47, 127.9, 127.8, 127.7, 127.4, 127.35, 113.7, 113.2, 97.0, 96.6, 94.9, 92.5, 90.74, 90.68, 88.6, 74.5, 74.3, 70.7, 55.3, 32.1, 24.8. HRMS for C_51_H_46_BrFN_3_O_6_ (M + H). Calcd: *m*/*z* 894.2554, found: *m*/*z* 894.2541, 896.2532.

*4-Amino-1-((2R,3R,4R,5R)-5-(chloromethyl)-3-fluoro-4-hydroxy-5-(1-hydroxyethyl)tetrahydrofuran-2-yl)pyrimidin-2(1H)-one 25*: To a solution of **23** (150 mg, 0.177 mmol) in methanol (3 mL) was added sodium borohydride (35 mg, 0.9 mmol) portion wise. The mixture was stirred for 30 min at room temperature, quenched with water (20 mL) and extracted with dichloromethane (3 × 20 mL). The combined organic layers were dried over sodium sulfate, filtered and concentrated under reduced pressure. The resulting crude product was purified by flash column chromatography (Hexanes/Ethyl acetate, 100/0 to 33/67) to give the desired hydroxy intermediate (134 mg, 89%) as a 1:1 mixture of isomers. A solution of the mixture (134 mg, 0.159 mmol) in 80% acetic acid (10 mL) was heated at 60–65 °C overnight. The volatiles were then evaporated under reduced pressure and the residue was purified by flash column chromatography (dichloromethane/methanol, 100/0 to 90/10) to give **25** (42 mg, 88%) as a 1:1 mixture of isomers. ^1^H NMR (400 MHz, DMSO-d_6_): δ 7.88 (d, *J* = 7.4 Hz, 0.5H), 7.87 (d, *J* = 7.4 Hz, 0.5H), 7.39 (bs, 0.5H), 7.35 (bs, 0.5H), 7.31(bs, 0.5H), 7.29 (bs, 0.5H), 6.19 (dd, *J* = 13.1, 6.6 Hz, 0.5H), 6.07 (dd, *J* = 16.2, 4.2 Hz, 0.5H), 5.95 (bs, 0.5H), 5.88 (bs, 0.5H), 5.81 (d, *J* = 7.4 Hz, 0.5H), 5.76 (d, *J* = 7.4 Hz, 0.5H), 5.50 (bs, 0.5H), 5.11 (m, 1H), 4.51 (dd, *J* = 13.8, 5.3 Hz, 0.5H), 4.37 (bs, 0.5H), 4.05 (m, 1H), 3.91 (d, *J* = 11.0 Hz, 0.5H), 3.85 (d, *J* = 12.8 Hz, 0.5H), 3.78 (d, *J* = 12.7 Hz, 0.5H), 1.15 (d, *J* = 6.6 Hz, 1.5H), 1.08 (d, *J* = 6.4 Hz, 1.5H). ^19^F NMR (376 MHz, DMSO-d_6_): δ −199.2 (dt, *J* = 57.5, 15.3 Hz), −206.2 (dd, *J* = 57.5, 13.7 Hz). ^13^C NMR (101 MHz, DMSO-d_6_): δ 166.2, 166.1, 155.6, 142.4, 142.0, 95.6, 95.0, 94.7, 93.5, 92.9, 91.6, 88.6, 88.5, 87.7, 87.3, 86.1, 85.8, 70.2, 70.1, 86.4, 68.3, 67.2, 66.6, 47.5, 41.1, 18.5, 17.4. HRMS for C_11_H_16_ClFN_3_O_4_ (M + H). Calcd: *m*/*z* 308.0813, found: *m*/*z* 308.0804, 310.0773.

*4-Amino-1-((2R,3R,4R,5R)-5-(bromomethyl)-3-fluoro-4-hydroxy-5-(1-hydroxyethyl) tetrahydrofuran-2-yl)pyrimidin-2(1H)-one 26*: Title compound **26** was obtained as a 1:1 mixture of isomers from **24** using the same procedure as for compound **25**. Yield: 48% over two steps. ^1^H NMR (400 MHz, DMSO-d_6_) δ 7.876 (d, *J* = 7.4 Hz, 0.5H), 7.872 (d, *J* = 7.4 Hz, 0.5H), 7.32 (m, 2H), 6.2 (dd, *J* = 12.9, 6.7 Hz, 0.5H), 6.05 (dd, *J* = 16.5, 4.0 Hz, 0.5H), 5.94 (d, *J* = 5.4 Hz, 0.5H), 5.88 (d, *J* = 6.2 Hz, 0.5H), 5.80 (d, *J* = 7.4 Hz, 0.5H), 5.75 (d, *J* = 7.4 Hz, 0.5H), 5.49 (d, *J* = 5.4 Hz, 0.5H), 5.30 (d, *J* = 4.3 Hz, 0.5H), 5.10 (m, 1H), 4.54 (m, 0.5H), 4.36 (m, 0.5H), 4.129 (m, 0.5H), 4.04 (m, 0.5H), 3.75 (d, *J* = 10.1 Hz, 0.5H), 3.74 (d, *J* = 12.0 Hz, 0.5H), 3.65 (d, *J* = 12.0 Hz, 0.5H), 1.14 (d, *J* = 6.6 Hz, 1.5H), 1.07 (d, *J* = 6.4 Hz, 1.5H). ^19^F NMR (376 MHz, DMSO-d_6_): δ −198.1 (dt, *J* = 58.1, 16.2 Hz), -206.2 (dd, *J* = 57.5, 13.4 Hz). ^13^C NMR (101 MHz, DMSO-d_6_): δ 166.2, 166.1, 155.60, 155.58, 142.4, 142.0, 95.6, 95.0, 93.6, 93.1, 91.7, 88.00, 88.99, 87.8, 87.7, 87.3, 86.0, 85.7, 70.3, 70.2, 68.4, 68.29, 68.27, 67.2, 37.4, 32.2, 18.3, 17.3. HRMS for C_11_H_16_BrFN_3_O_4_ (M + H). Calcd: *m*/*z* 352.0308, found: *m*/*z* 352.0301, 354.0276.

### 3.2. Antiviral Activity Assays

#### 3.2.1. RSV Replicon Assay

RSV replicon cell lines were obtained from Apath, LLC (Brooklyn, NY, NY, USA) and were cultured as previously described [15]. Ribavirin and ALS-8112, synthesized by following reported procedures [11] were used as positive controls. Compounds were dissolved in dimethyl sulfoxide (DMSO) to a 40 mM concentration and serially diluted to the desired compound concentrations. Anti-viral activity was measured after 72 h by using Renilla-Glo reagent kit (Promega, Madison, WI, USA), according to manufacturer’s instruction.

#### 3.2.2. Zika Virus (ZIKV)

Human hepatoma (Huh7) cells were exposed to the newly synthesized drugs or 7-deaza-7-fluoro-2′-C-methyl adenosine (positive control) at concentrations up to 20 µM immediately following infection with ZIKV (multiplicity of infection, MOI = 0.5) Puerto Rican strain (PRVABC59) to assess antiviral activity. Cell cytopathic effect (CPE) MTS assay (Promega, Madison, WI, USA) was measured five days after compound addition to determine the levels of replication inhibition [16,17].

#### 3.2.3. Dengue Virus serotype 2 (DENV-2), West Nile Virus (WNV) or Chikungunya (CHIKV)

DENV2 or WNV replicon-harboring baby hamster kidney (BHK) cells and CHIKV replicon-harboring Huh7 cells were exposed to the newly synthesized drugs or 7-deaza-7-fluoro-2′-C-methyl adenosine or β-D-*N*^4^-hydroxycytidine (positive controls) at concentrations up to 30 µM to assess antiviral activity. Renilla luciferase levels (Promega, Madison, WI, USA) were quantified 48 h after test compounds addition to determine the levels of replication inhibition (EC_50_, µM) [18].

### 3.3. Toxicity Assays

Cytotoxicity assays. In vitro cytotoxicity was determined using the CellTiter 96 non-radioactive cell proliferation colorimetric assay (MTT assay, Promega, Madison, WI, USA) in primary human peripheral blood mononuclear (PBM) cells, human T lymphoblast (CEM) and human hepatocellular carcinoma (HepG2 or Huh7) cell lines. Toxicity levels were measured as the concentration of test compound that inhibited cell proliferation by 50% (CC_50_).

### 3.4. Crystallography

Single colorless plate crystals of compound **17** were recrystallised from methanol by slow evaporation. A suitable crystal with dimensions 0.41 × 0.30 × 0.15 mm^3^ was selected and mounted on a loop with paratone oil on a XtaLAB Synergy-S diffractometer (Rigaku Oxford Diffraction, Wroclaw, Poland). The crystal was kept at a steady T = 99.9(4) K during data collection. The structure was solved with the ShelXT [19] solution program using dual-space recycling methods and by using Olex2 (V1.3-alpha) [20] as the graphical interface. The model was refined with ShelXL 2018/3 [21] using full matrix least squares minimization on F2. Results from X-ray structure determination of 17 are the following. Crystal data for C_12_H_16_FN_3_O_5_, *M_r_* = 301.28, orthorhombic, *P*2_1_2_1_2_1_ (No. 19), a = 7.3409(5) Å, b = 8.2950(5) Å, c = 19.9788(15) Å, *α* = *β* = *γ* = 90°, *V* = 1216.56(14) Å^3^, *T* = 99.9(4) K, *Z* = 4, *Z*′ = 1, *µ*(Cu K*_α_*) = 1.192, 11534 reflections measured, 2161 unique (*R_int_* = 0.0568) which were used in all calculations. The final *wR*_2_ was 0.1111 (all data) and *R*_1_ was 0.0417 (I > 2σ(I)). (More details available in Supplementary Materials.)

## 4. Conclusions

Based on the structure of anti-RSV agent ALS-8112, a series of 4′- and 5′- substituted-2′-deoxy-2′-fluoro cytidine nucleoside analogs were synthesized in 10 to 13 steps from commercially available 2′-deoxy-2′-α-fluorocytidine. Nucleosides analogs with an azetidine, an oxetane, and an isoxazole ring, as well as a difluoromethyl ether group, four groups never previously introduced at the 4′-position of a nucleoside, were successfully prepared. Interestingly, the formation of the 4′-oxetane ring via a Johnson–Corey–Chaykovsky epoxidation and consecutive ring-expansion was completely stereoselective, as determined by single crystal X-ray diffraction. We hypothesized that this selectivity could be attributed to the monomethoxytrityl groups hindering one face of the molecule. Final 4′- and 5′-substituted nucleosides (11, 14, 17, 20, 25, and 26) were evaluated for antiviral activity but unfortunately, none of them showed marked activity when tested against RSV, ZIKV, DENV-2, WNV, or CHIKV.

## Supplementary Materials

The following are available online. Figures S1–S18: ^1^H, ^13^C and ^19^F-NMR spectra for compounds **11, 14, 17, 20, 25** and **26**, Table S1: Fractional Atomic Coordinates (×10^4^) and Equivalent Isotropic Displacement Parameters (Å^2^ × 10^3^) for **14**. U_eq_ is defined as 1/3 of the trace of the orthogonalised U_ij_; Table S2: Anisotropic Displacement Parameters (×104) for **14**. The anisotropic displacement factor exponent takes the form: −2π^2^[h^2^a*^2^ × U_11_+ ... +2hka* × b* × U_12_]; Table S3: Bond Lengths in Å for 14; Table S4: Bond Angles in ° for **14**; Table S5: Torsion Angles in ° for **14**; Table S6: Hydrogen Fractional Atomic Coordinates (×10^4^) and Equivalent Isotropic Displacement Parameters (Å^2^ × 10^3^) for **14**. U_eq_ is defined as 1/3 of the trace of the orthogonalised U_ij_.
