# Supplementary material for: Synthesis of 4′-Substituted-2′-Deoxy-2′-α-Fluoro Nucleoside Analogs as Potential Antiviral Agents
Source: Molecules. 2020 Mar 11;25(6):1258. doi: 10.3390/molecules25061258 (PMC7143966; doi:10.3390/molecules25061258)
Supplement: Supplementary file 1 [file molecules-25-01258-s001.pdf]

Supporting Information

Synthesis of 4'-Substituted-2'-Deoxy-2'-α-Fluoro Nucleoside Analogs as Potential Antiviral Agents

Mahesh Kasthuri, Chengwei Li, Kiran Verma, Olivia Ollinger Russell, Lyndsey Dickson, Louise McCormick, Leda Bassit, Franck Amblard, Raymond F. Schinazi*

[1] Center for AIDS Research, Laboratory of Biochemical Pharmacology, Department of Pediatrics, Emory University School of Medicine, 1760 Haygood Drive, Atlanta, GA 30322, USA;

***** Correspondence: rschina@emory.edu

Table of Contents:

1. ^{1}H, ^{13}C and ^{19}F-NMR spectra for compounds **11, 14, 17, 20, 25** and **26** S2-S19
2. Crystallographic data for compound **17** S20-25

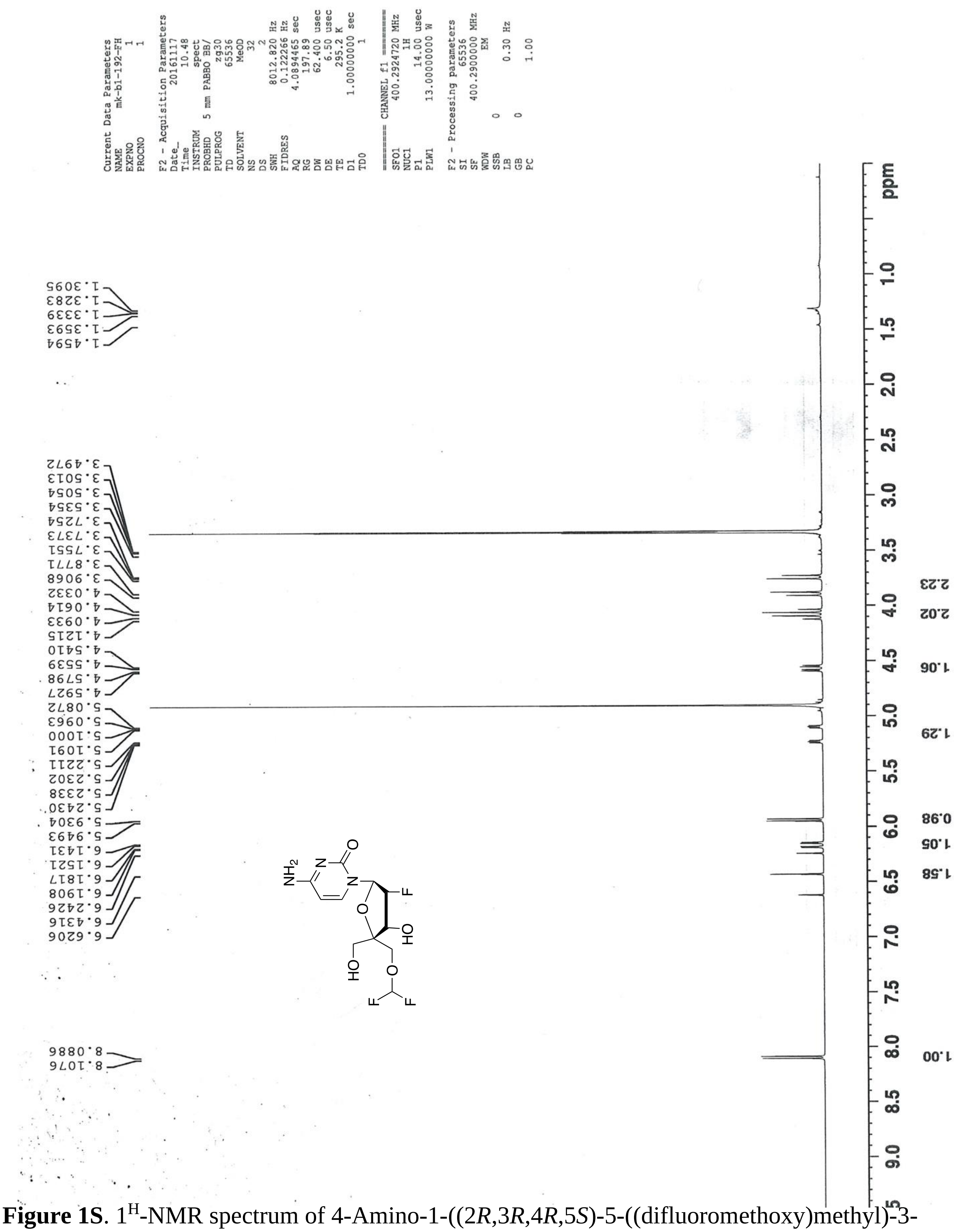

Figure 1S. 1^{H}-NMR spectrum of 4-Amino-1-((2*R*,3*R*,4*R*,5*S*)-5-((difluoromethoxy)methyl)-3-fluoro-4-hydroxy-5-(hydroxymethyl) tetrahydrofuran-2-yl)pyrimidin-2(1*H*)-one **(11)**

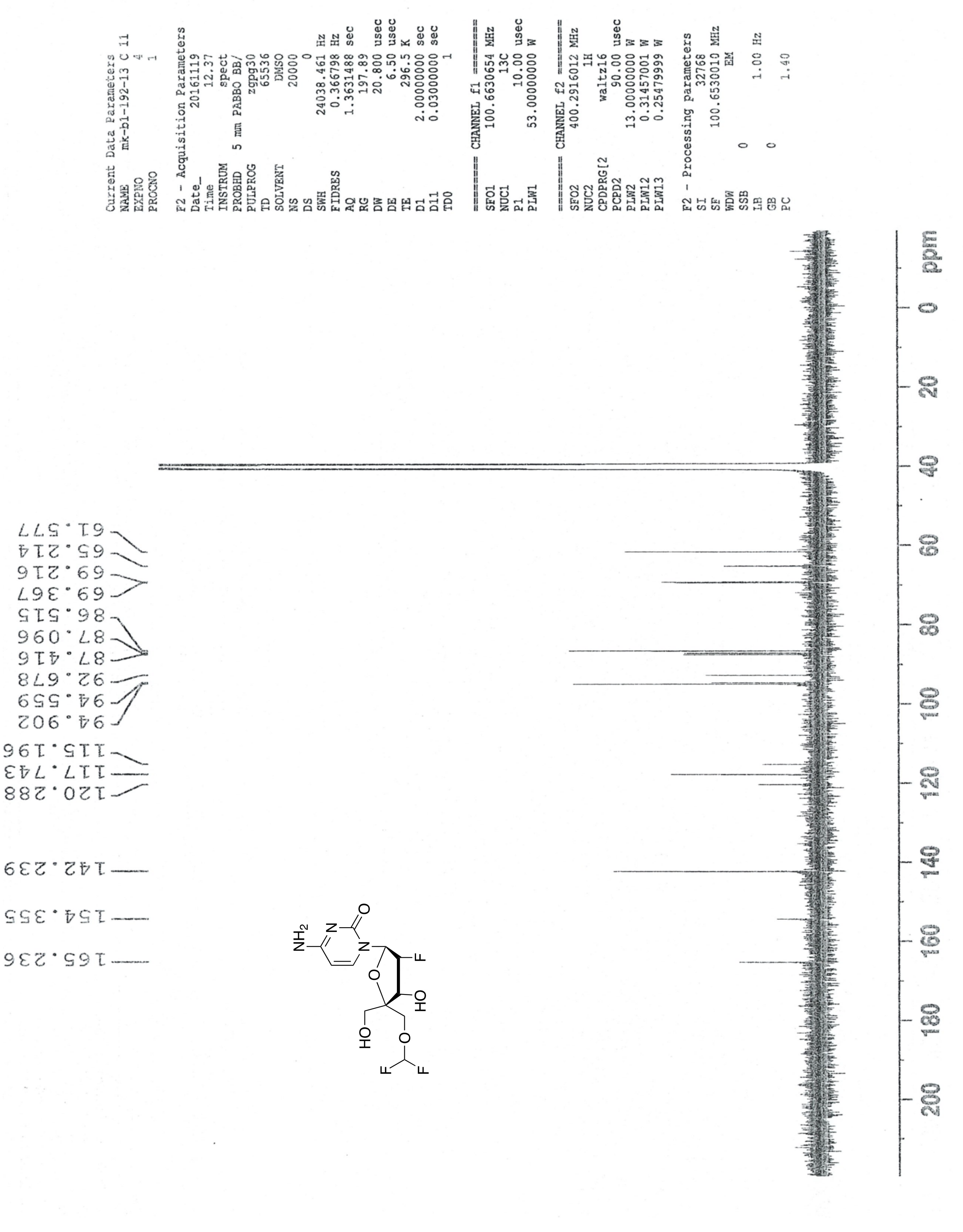

Figure 2S. ^{13}C-NMR spectrum of 4-Amino-1-((2*R*,3*R*,4*R*,5*S*)-5-((difluoromethoxy)methyl)-3-fluoro-4-hydroxy-5-(hydroxymethyl) tetrahydrofuran-2-yl)pyrimidin-2(1*H*)-one **(11)**

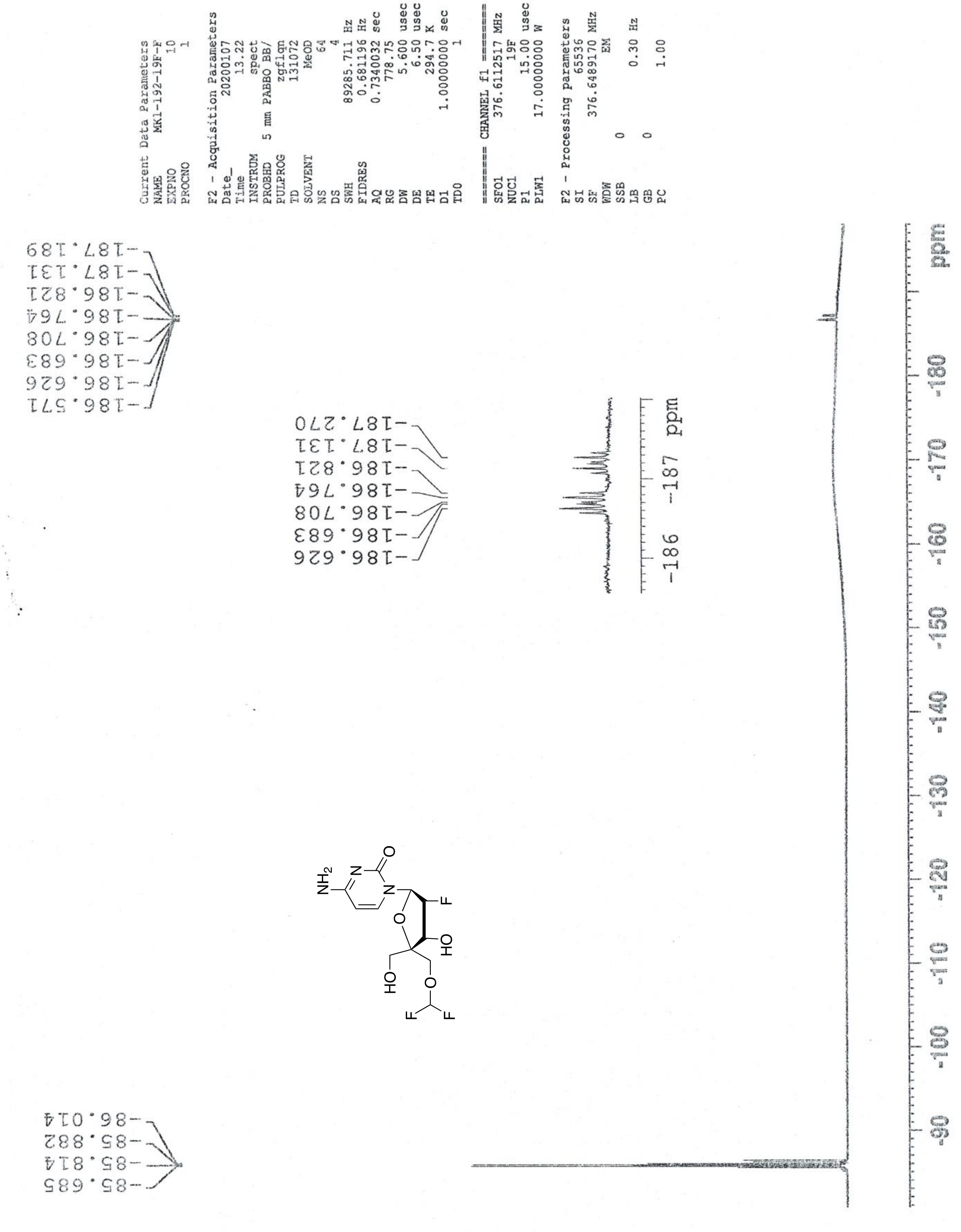

Figure 4S. ^{19}F-NMR spectrum of 4-Amino-1-((2*R*,3*R*,4*R*,5*S*)-5-((difluoromethoxy)methyl)-3-fluoro-4-hydroxy-5-(hydroxymethyl) tetrahydrofuran-2-yl)pyrimidin-2(1*H*)-one **(11)**

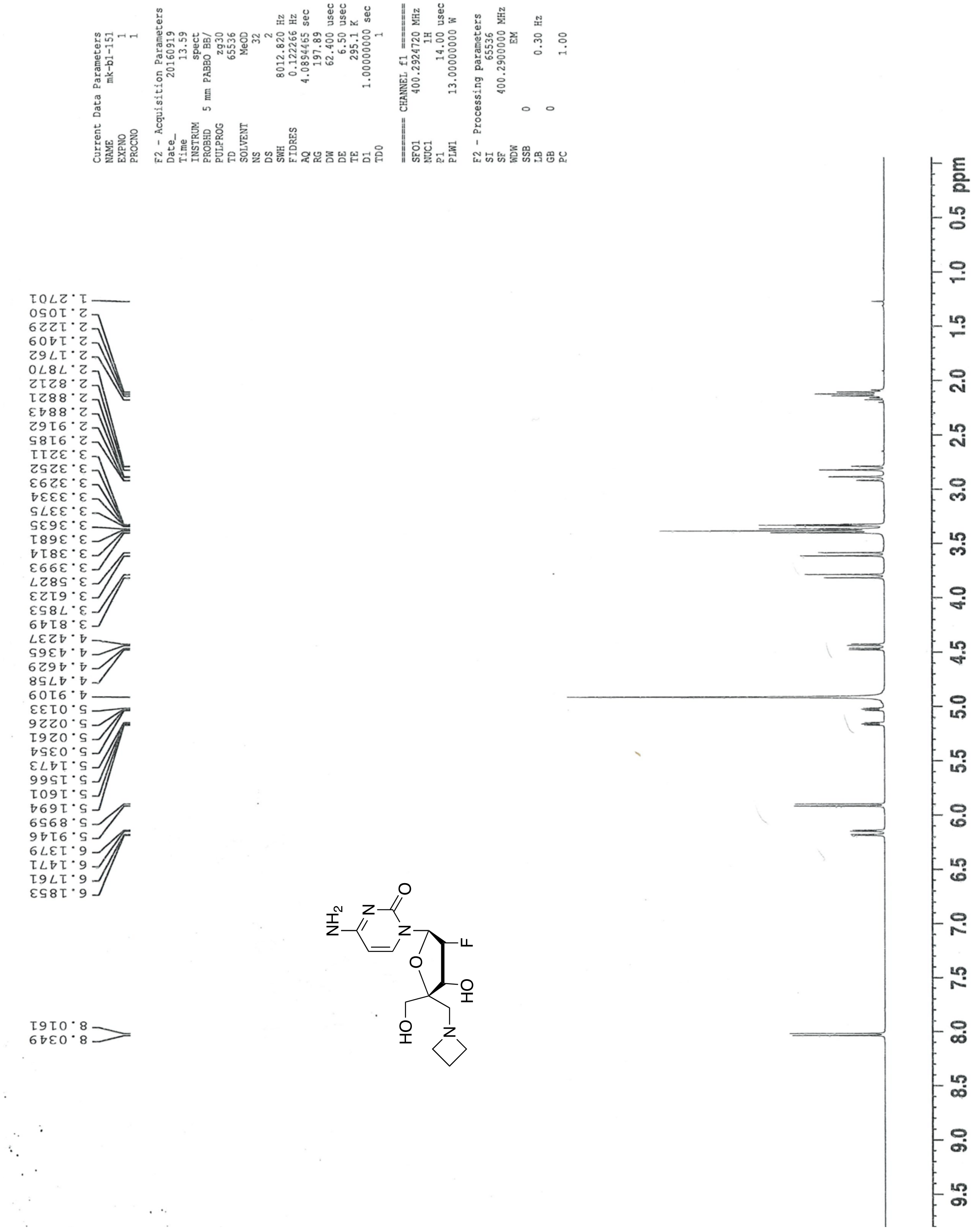

Figure 4S. ^{1}H-NMR spectrum of 4-Amino-1-((2*R*,3*R*,4*R*,5*R*)-5-(azetidin-1-ylmethyl)-3-fluoro-4-hydroxy-5-(hydroxymethyl) tetrahydrofuran-2-yl)pyrimidin-2(1*H*)-one **(14)**

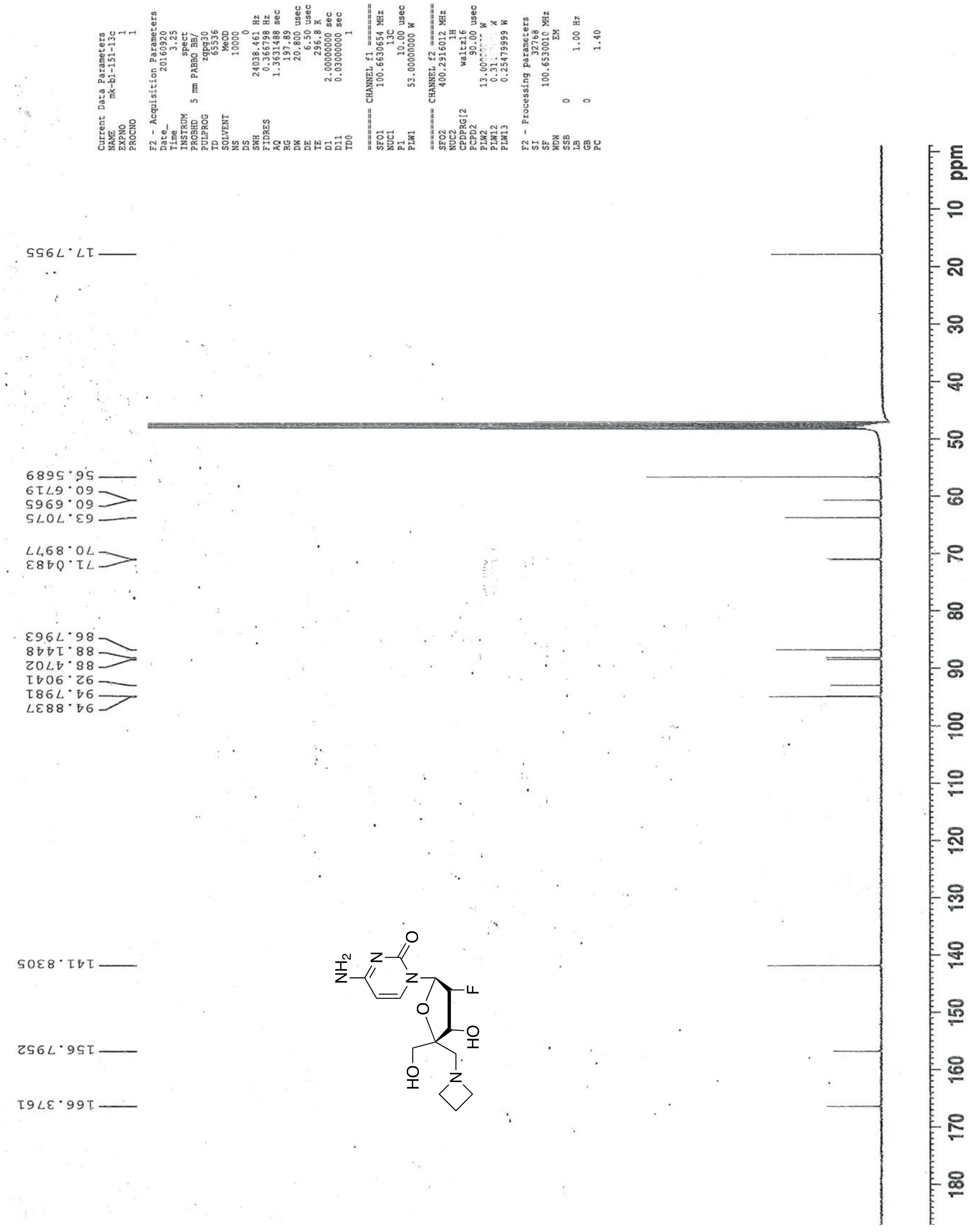

Figure 5S. ^{13}C-NMR spectrum of 4-Amino-1-((2*R*,3*R*,4*R*,5*R*)-5-(azetidin-1-ylmethyl)-3-fluoro-4-hydroxy-5-(hydroxymethyl) tetrahydrofuran-2-yl)pyrimidin-2(1*H*)-one **(14)**

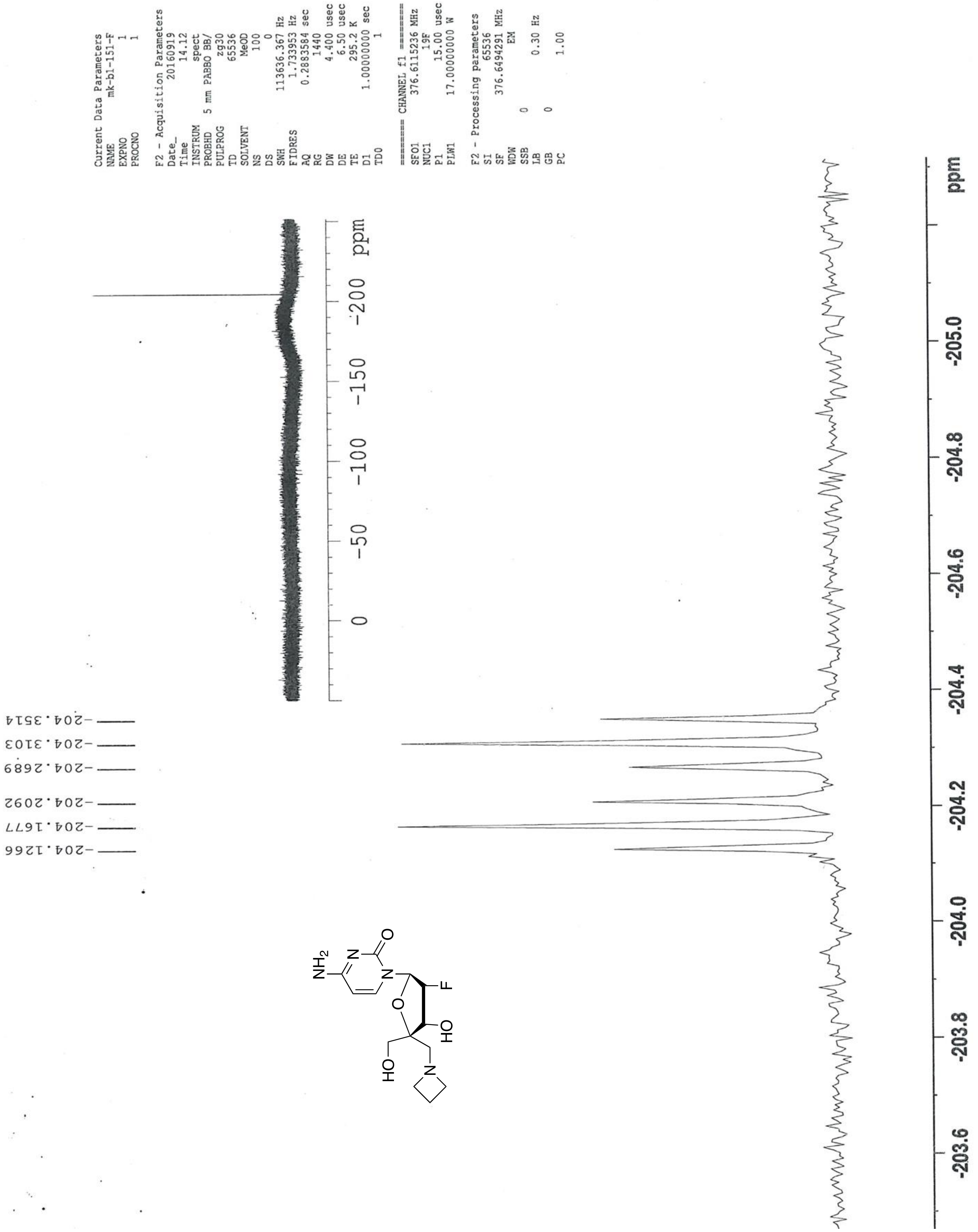

Figure 6S. ^{19}F-NMR spectrum of 4-Amino-1-((2*R*,3*R*,4*R*,5*R*)-5-(azetidin-1-ylmethyl)-3-fluoro-4-hydroxy-5-(hydroxymethyl) tetrahydrofuran-2-yl)pyrimidin-2(1*H*)-one **(14)**

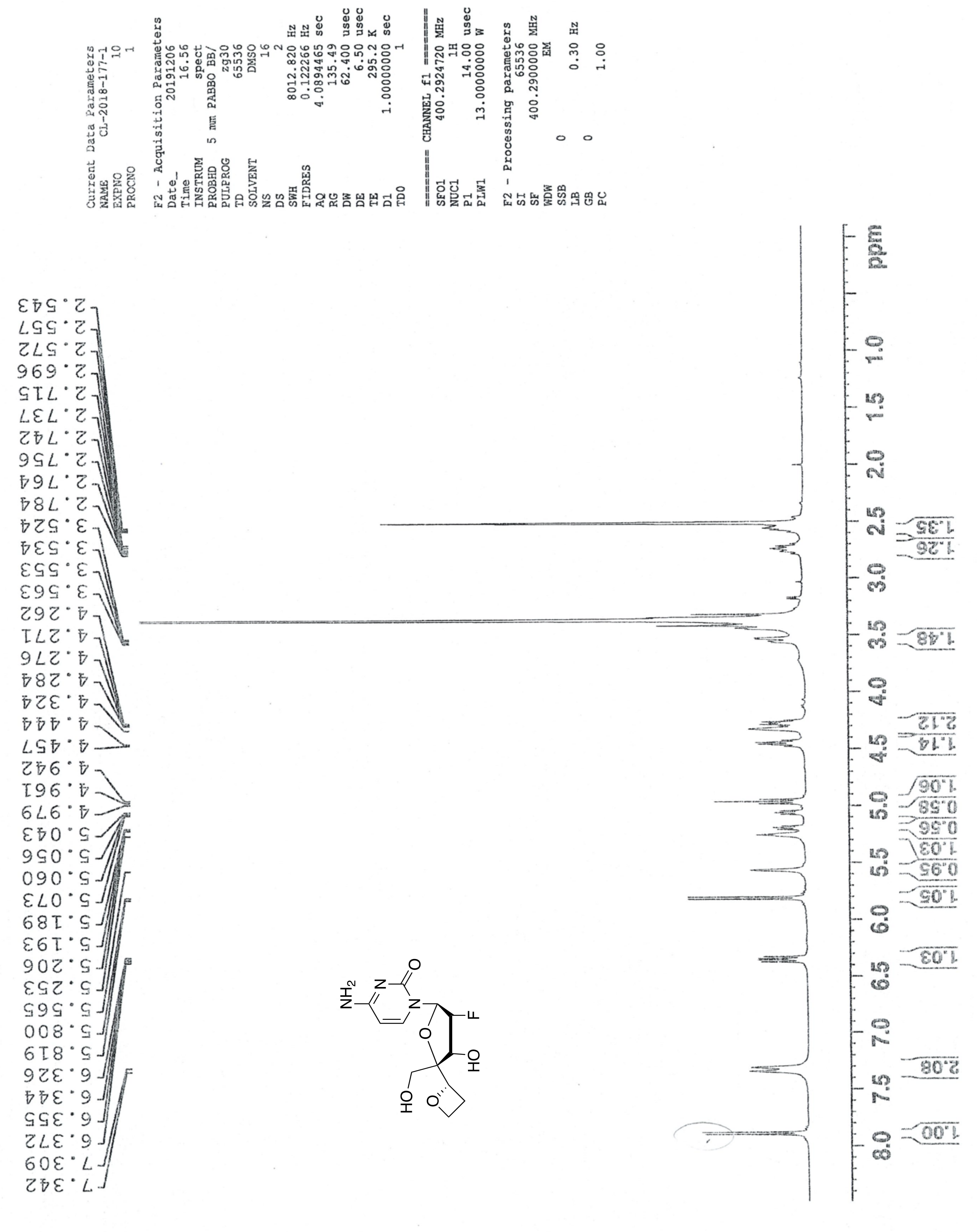

Figure 7S. ^{1}H-NMR spectrum of 4-Amino-1-((2*R*,3*R*,4*R*,5*R*)-3-fluoro-4-hydroxy-5-(hydroxymethyl)-5-((S)-oxetan-2-yl) tetrahydrofuran-2-yl)pyrimidin-2(1*H*)-one **(17)**

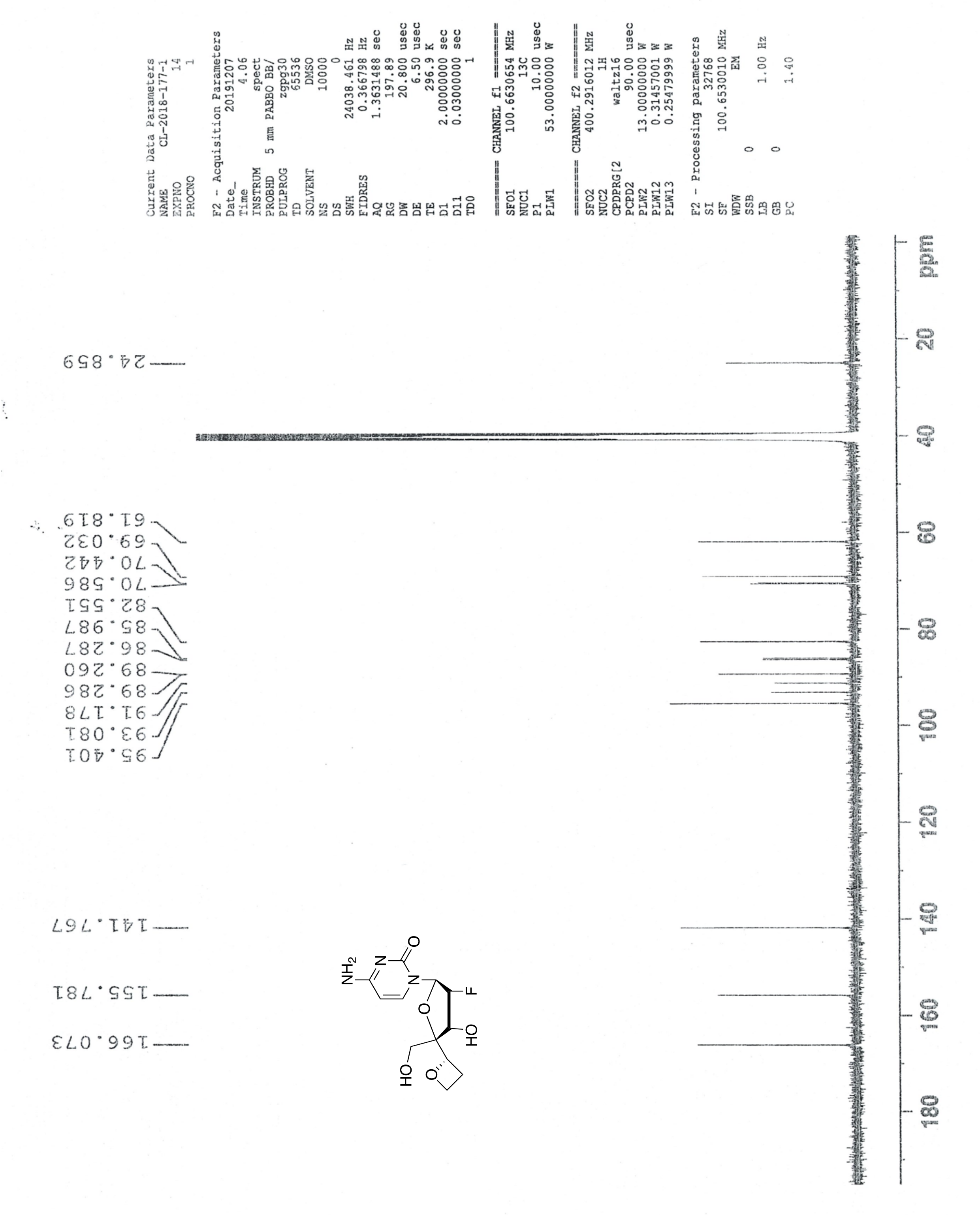

Figure 8S. ^{13}C-NMR spectrum of 4-Amino-1-((2*R*,3*R*,4*R*,5*R*)-3-fluoro-4-hydroxy-5-(hydroxymethyl)-5-((S)-oxetan-2-yl) tetrahydrofuran-2-yl)pyrimidin-2(1*H*)-one **(17)**

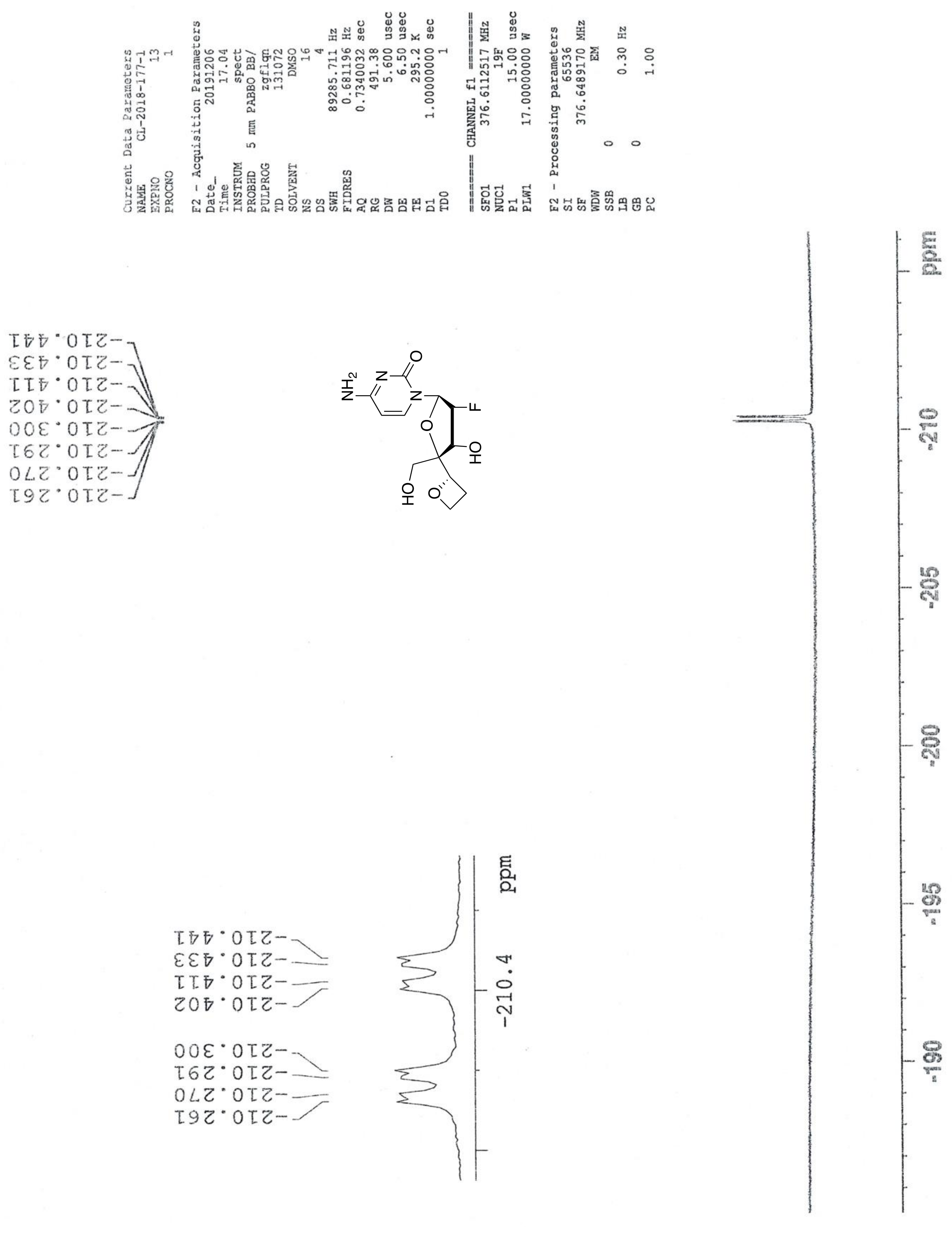

Figure 9S. ^{19}F-NMR spectrum of 4-Amino-1-((2*R*,3*R*,4*R*,5*R*)-3-fluoro-4-hydroxy-5-(hydroxymethyl)-5-((S)-oxetan-2-yl) tetrahydrofuran-2-yl)pyrimidin-2(1*H*)-one **(17)**

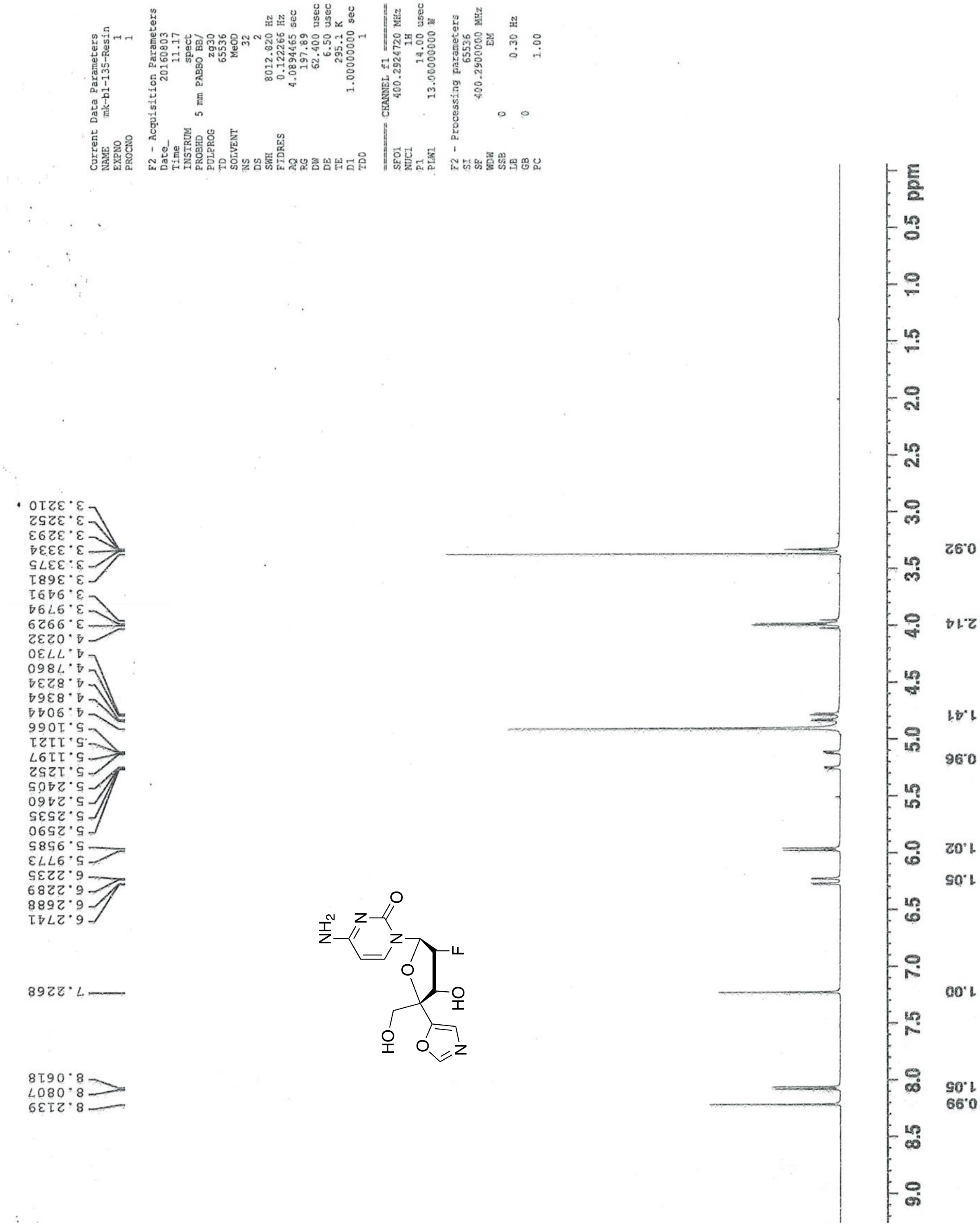

Figure 10S. ^{1}H-NMR spectrum of 4-Amino-1-((2*R*,3*R*,4*R*,5*R*)-3-fluoro-4-hydroxy-5-(hydroxymethyl)-5-(oxazol-5-yl)tetrahydrofuran-2-yl)pyrimidin-2(1*H*)-one **(20)**

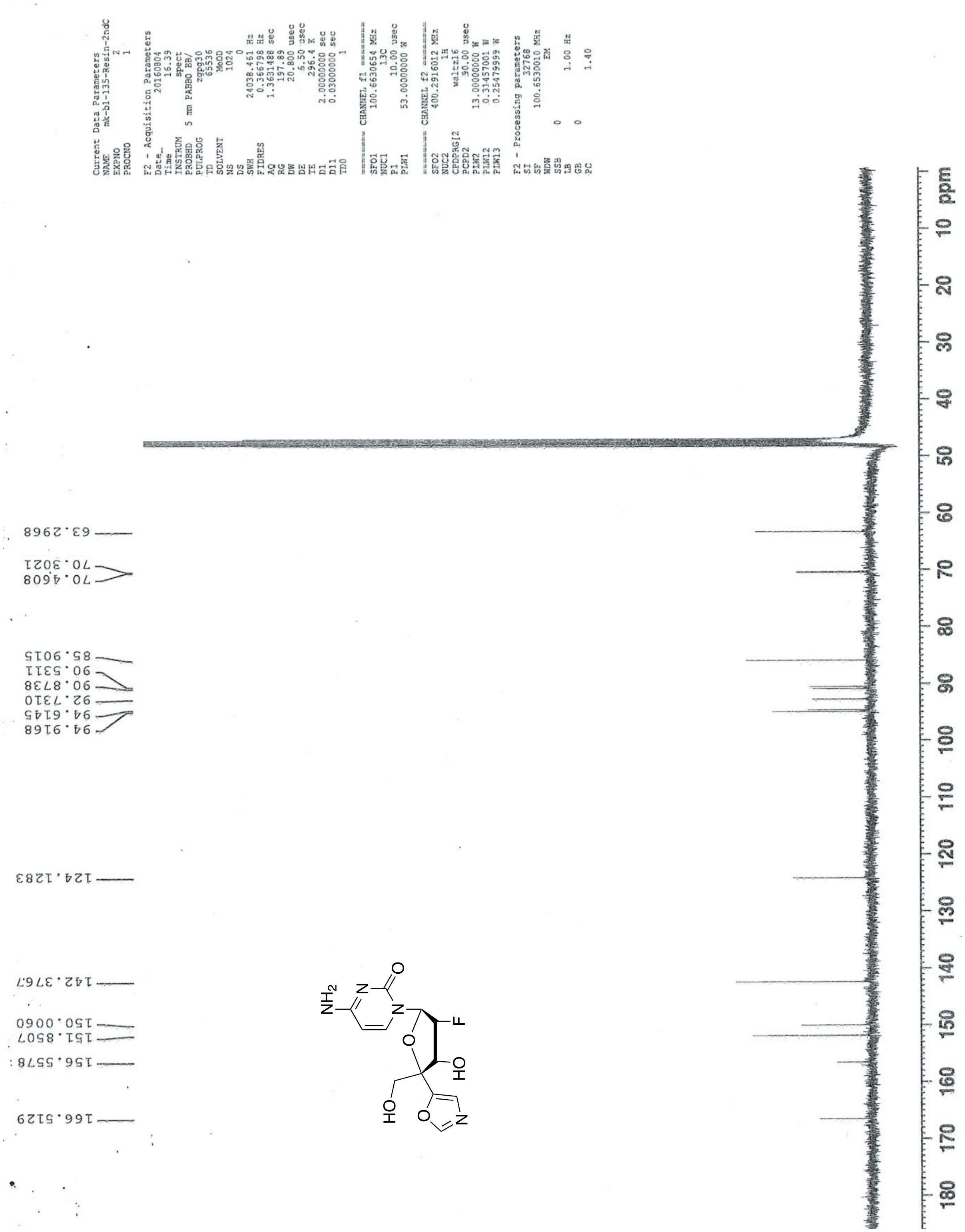

Figure 11S. ^{13}C-NMR spectrum of 4-Amino-1-((2*R*,3*R*,4*R*,5*R*)-3-fluoro-4-hydroxy-5-(hydroxymethyl)-5-(oxazol-5-yl)tetrahydrofuran-2-yl)pyrimidin-2(1*H*)-one **(20)**

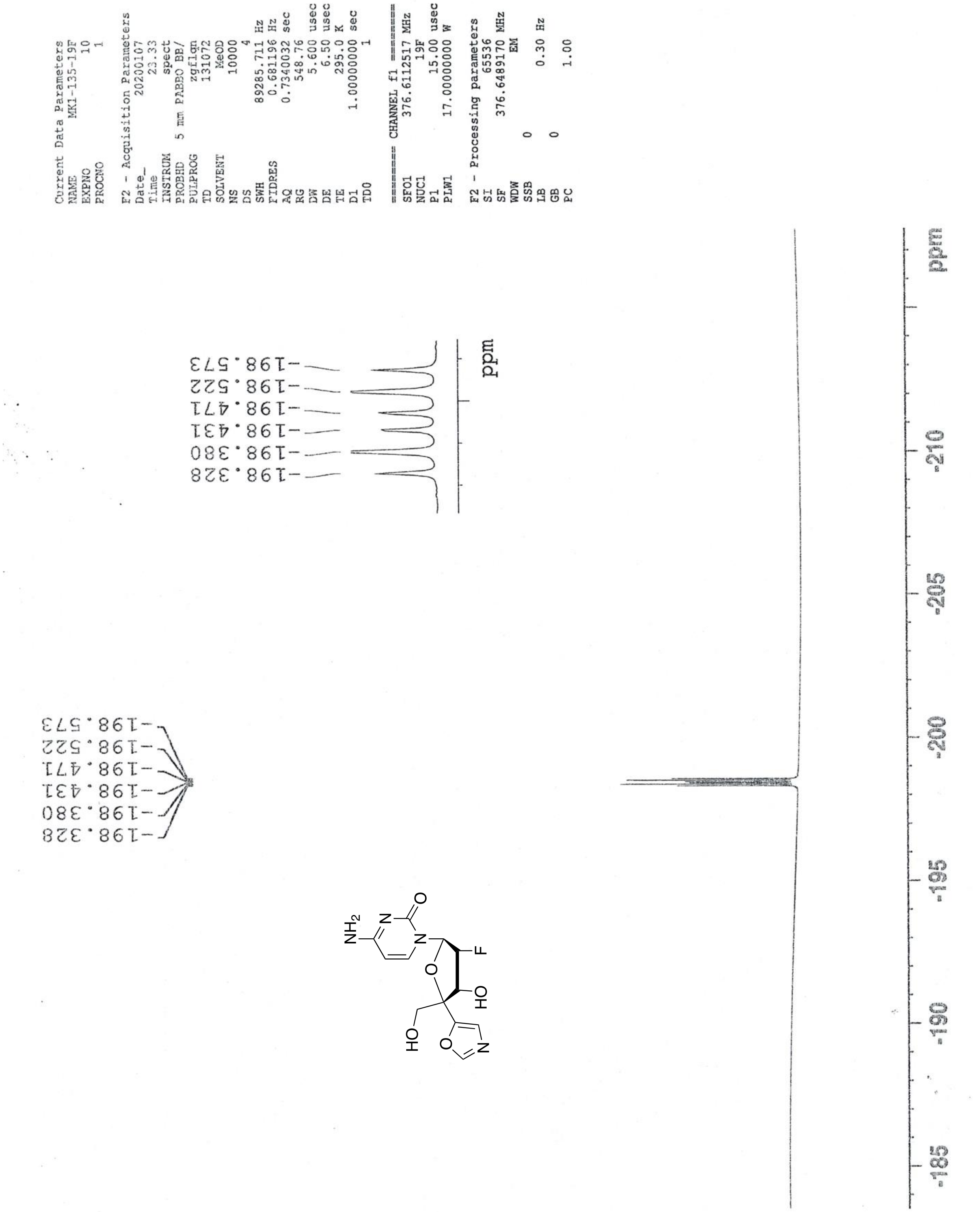

Figure 12S. ^{19}F-NMR spectrum of 4-Amino-1-((2*R*,3*R*,4*R*,5*R*)-3-fluoro-4-hydroxy-5-(hydroxymethyl)-5-(oxazol-5-yl)tetrahydrofuran-2-yl)pyrimidin-2(1*H*)-one **(20)**

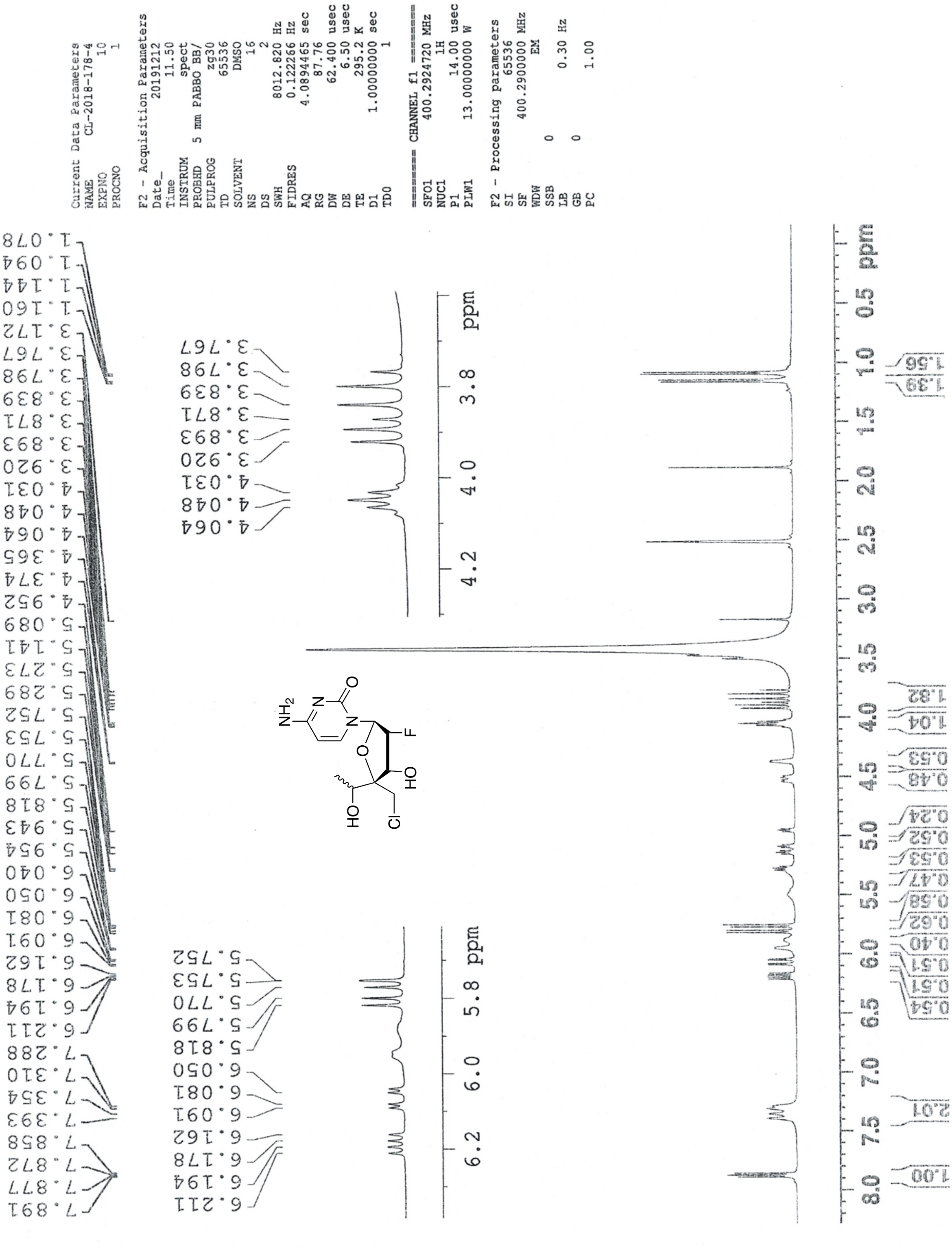

Figure 13S. ^{1}H-NMR spectrum of 4-Amino-1-((2*R*,3*R*,4*R*,5*R*)-5-(chloromethyl)-3-fluoro-4-hydroxy-5-(1-hydroxyethyl)tetrahydrofuran-2-yl)pyrimidin-2(1*H*)-one **(25)**

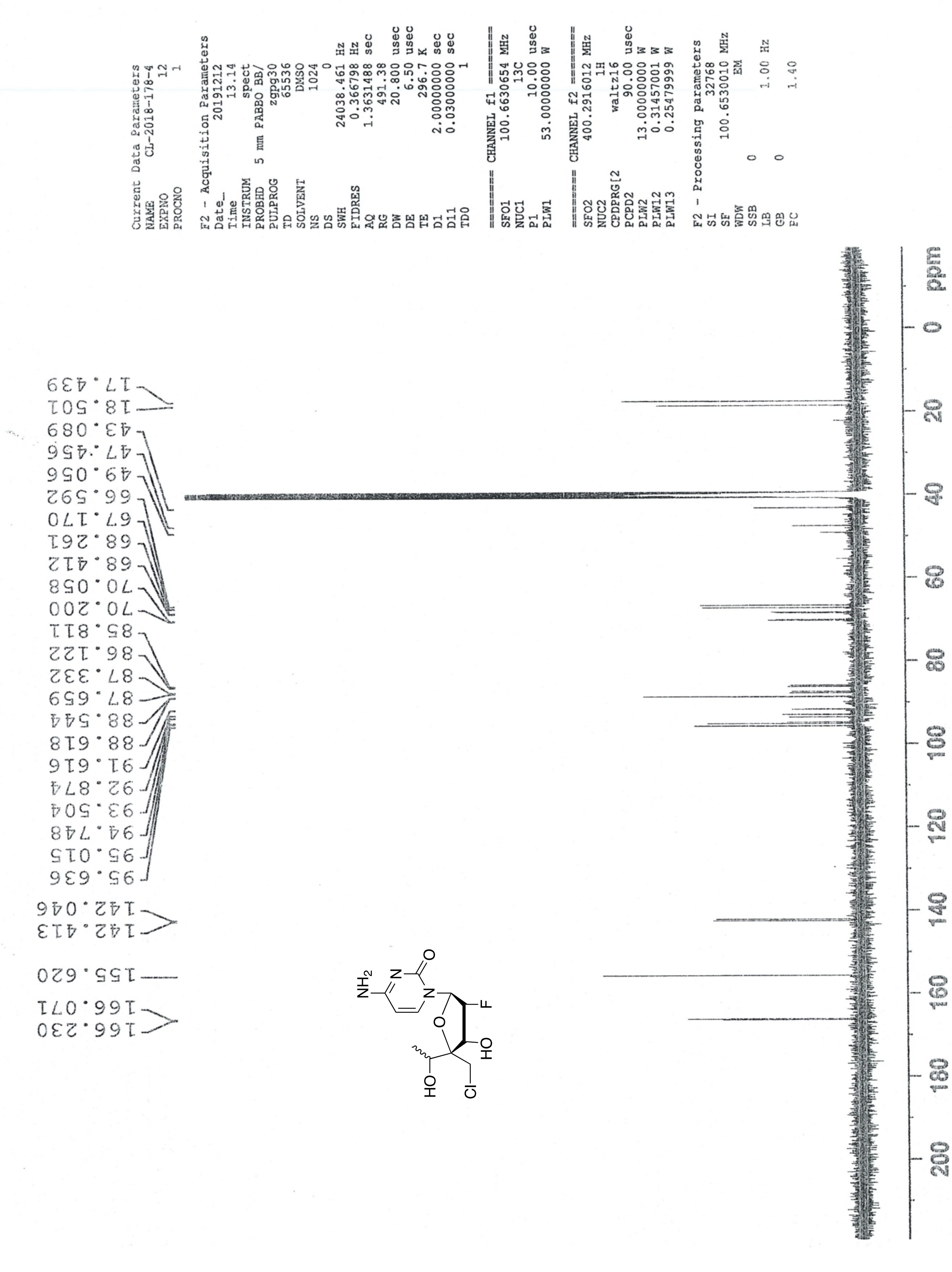

Figure 14S. ^{13}C-NMR spectrum of 4-Amino-1-((2*R*,3*R*,4*R*,5*R*)-5-(chloromethyl)-3-fluoro-4-hydroxy-5-(1-hydroxyethyl)tetrahydrofuran-2-yl)pyrimidin-2(1*H*)-one **(25)**

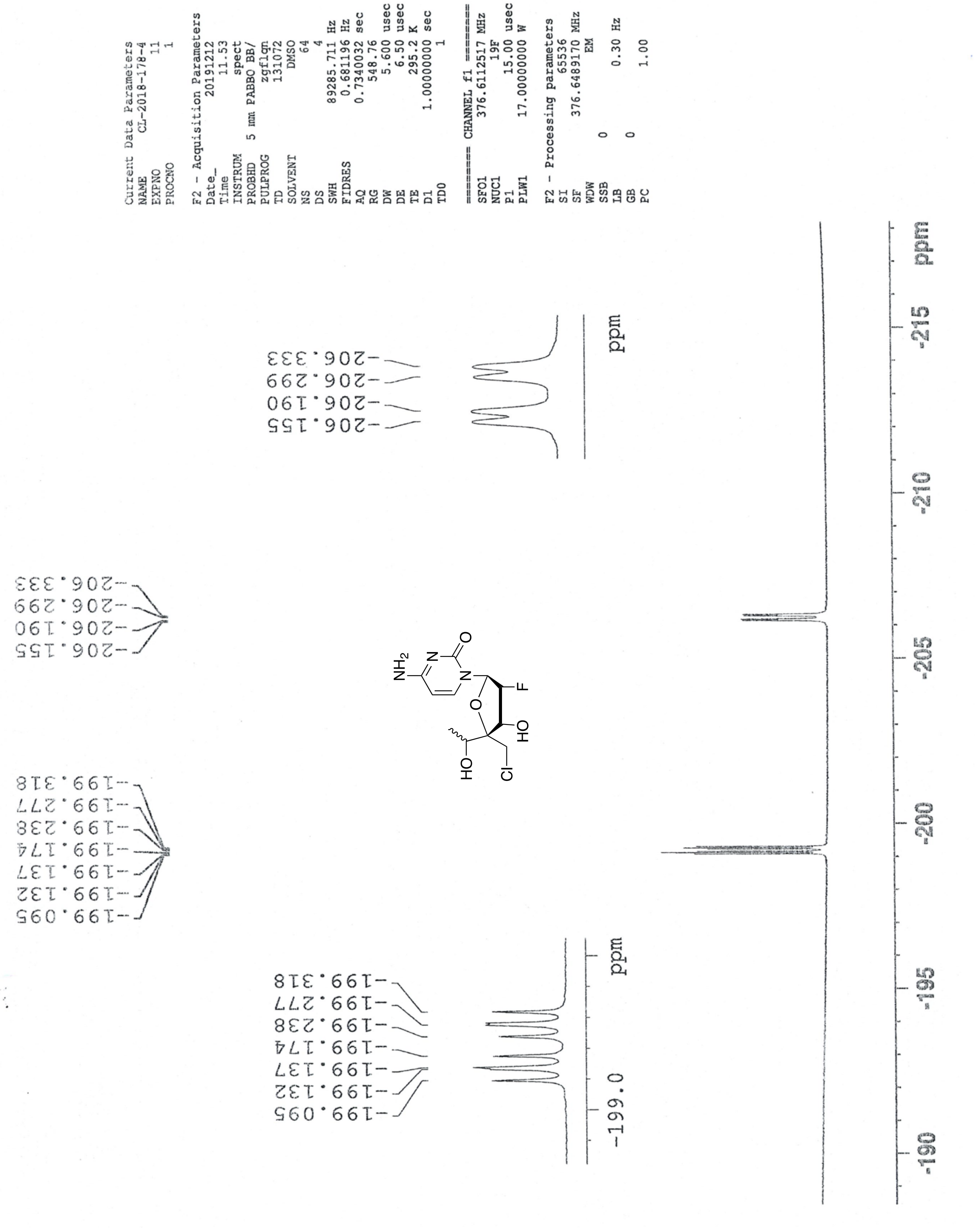

Figure 15S. ^{19}F-NMR spectrum of 4-Amino-1-((2*R*,3*R*,4*R*,5*R*)-5-(chloromethyl)-3-fluoro-4-hydroxy-5-(1-hydroxyethyl)tetrahydrofuran-2-yl)pyrimidin-2(1*H*)-one **(25)**

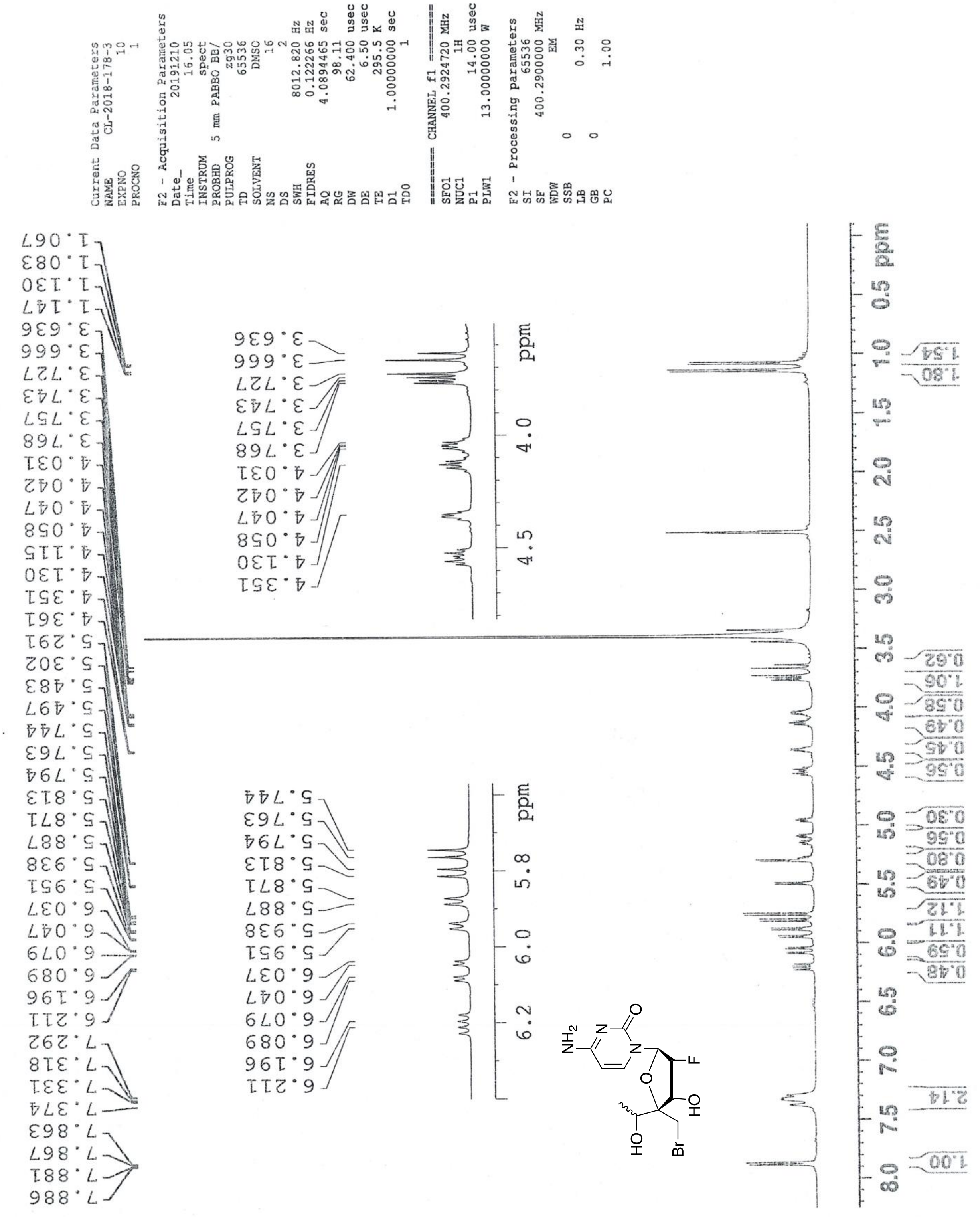

Figure 16S. ^{1}H-NMR spectrum of 4-Amino-1-((2*R*,3*R*,4*R*,5*R*)-5-(bromomethyl)-3-fluoro-4-hydroxy-5-(1-hydroxyethyl) tetrahydrofuran-2-yl)pyrimidin-2(1*H*)-one **(26)**

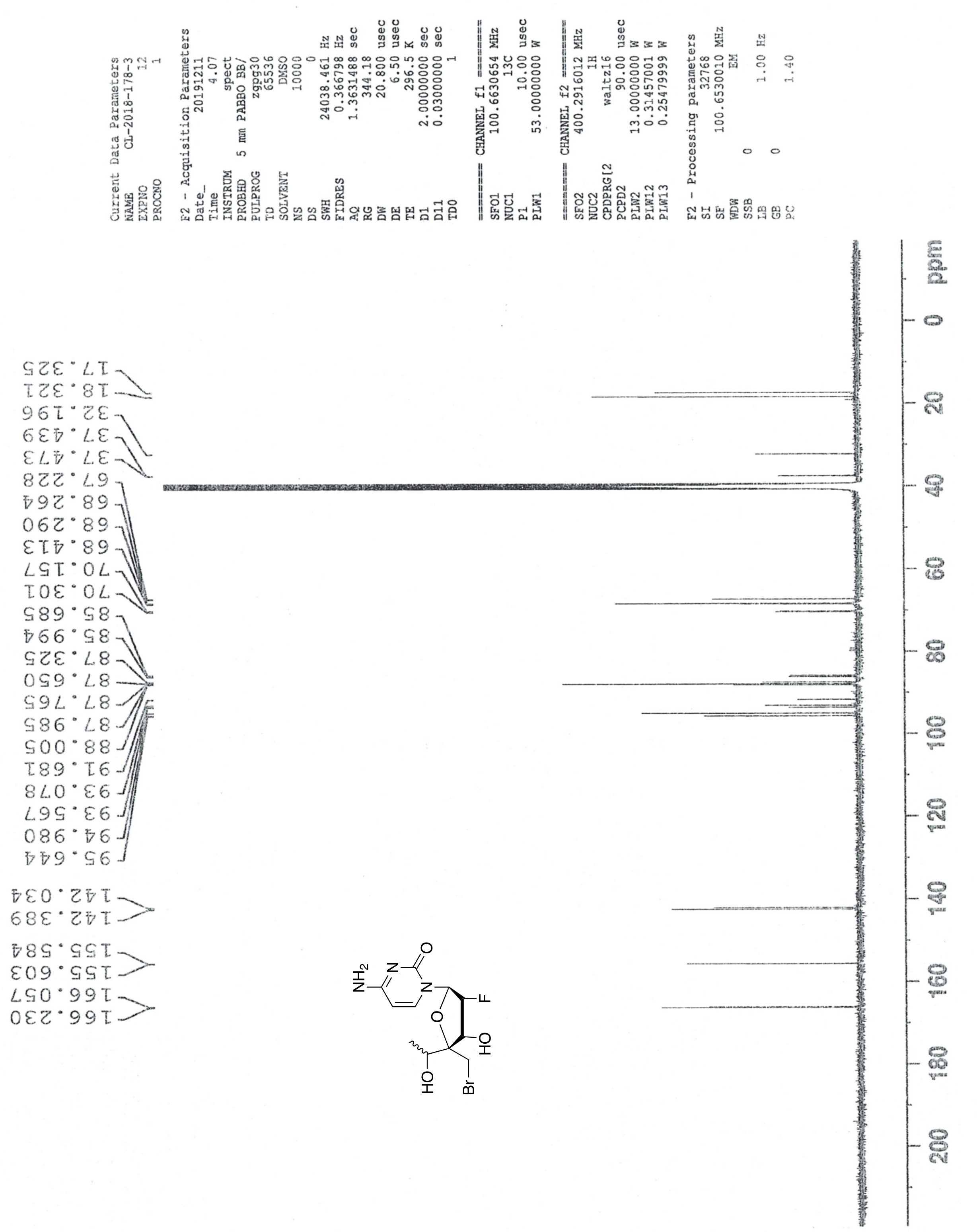

Figure 17S. ^{13}C-NMR spectrum of 4-Amino-1-((2*R*,3*R*,4*R*,5*R*)-5-(bromomethyl)-3-fluoro-4-hydroxy-5-(1-hydroxyethyl) tetrahydrofuran-2-yl)pyrimidin-2(1*H*)-one **(26)**

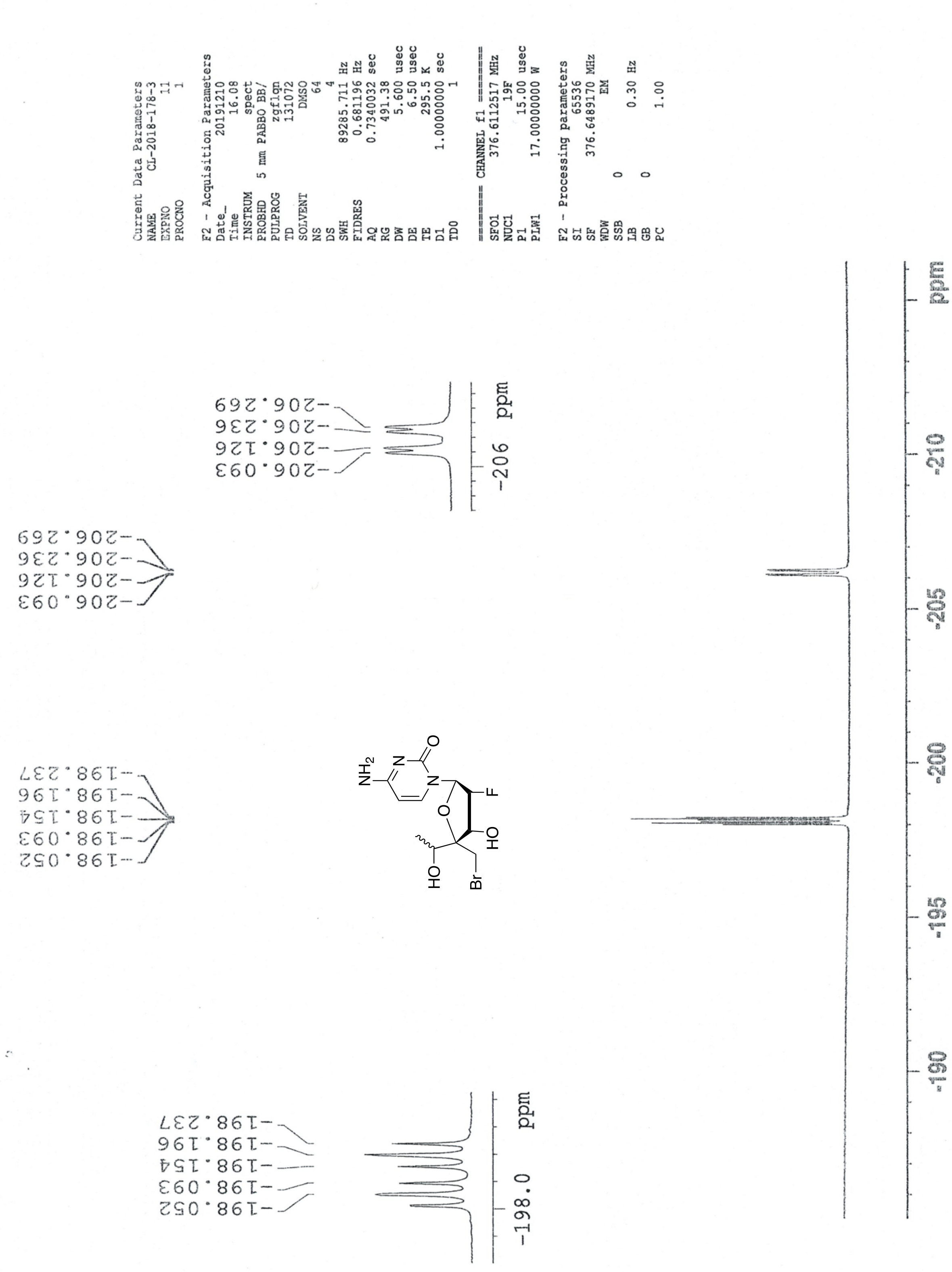

Figure 18S. ^{19}F-NMR spectrum of 4-Amino-1-((2*R*,3*R*,4*R*,5*R*)-5-(bromomethyl)-3-fluoro-4-hydroxy-5-(1-hydroxyethyl) tetrahydrofuran-2-yl)pyrimidin-2(1*H*)-one **(26)**

Crystal Data and Experimental

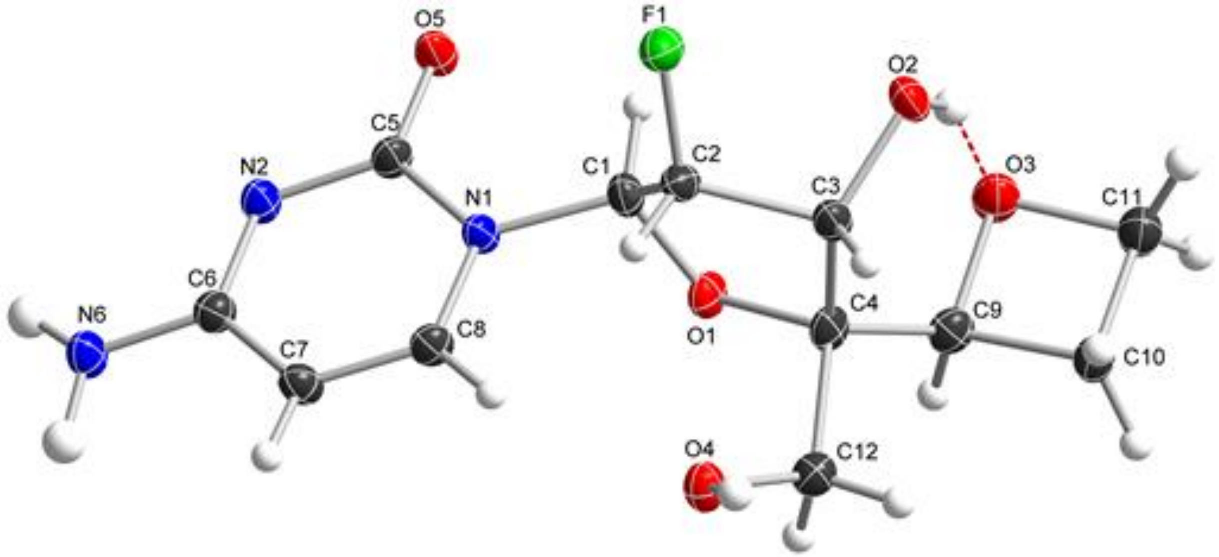

Experimental. Single colourless plate crystals of **17** were recrystallised from methanol by slow evaporation. A suitable crystal with dimensions 0.41 × 0.30 × 0.15 mm^3 was selected and mounted on a loop with paratone oil on a XtaLAB Synergy-S diffractometer. The crystal was kept at a steady T = 99.9(4) K during data collection. The structure was solved with the ShelXT (Sheldrick, 2015) solution program using dual-space recycling methods and by using **Olex2** (Dolomanov et al., 2009) as the graphical interface. The model was refined with ShelXL 2018/3 (Sheldrick, 2015) using full matrix least squares minimisation on F^2.

Crystal Data. $C_{12}H_{16}FN_3O_5$, M_r = 301.28, orthorhombic, $P2_12_12_1$ (No. 19), a = 7.3409(5) Å, b = 8.2950(5) Å, c = 19.9788(15) Å, $\alpha = \beta = \gamma$ = 90°, V = 1216.56(14) Å^3, T = 99.9(4) K, Z = 4, Z' = 1, μ(Cu K$_\alpha$) = 1.192, 11534 reflections measured, 2161 unique (R_{int} = 0.0568) which were used in all calculations. The final wR_2 was 0.1111 (all data) and R_1 was 0.0417 (I > 2σ(I)).

Formula	$C_{12}H_{16}FN_3O_5$
$D_{calc.}$/ g cm^{-3}	1.645
μ/mm^{-1}	1.192
Formula Weight	301.28
Colour	colourless
Shape	plate
Size/mm^3	0.41×0.30×0.15
T/K	99.9(4)
Crystal System	orthorhombic
Flack Parameter	-0.07(11)
Hooft Parameter	-0.02(7)
Space Group	$P2_12_12_1$
a/Å	7.3409(5)
b/Å	8.2950(5)
c/Å	19.9788(15)
α/°	90
β/°	90
γ/°	90
V/Å^3	1216.56(14)
Z	4
Z'	1
Wavelength/Å	1.54184
Radiation type	Cu K_α
Θ_{min}/°	4.426
Θ_{max}/°	67.041
Measured Refl's.	11534
Ind't Refl's	2161
Refl's with I > 2σ(I)	2062
R_{int}	0.0568
Parameters	200
Restraints	3
Largest Peak	0.327
Deepest Hole	-0.297
GooF	1.110
wR_2 (all data)	0.1111
wR_2	0.1027
R_1 (all data)	0.0440
R_1	0.0417

Table 1: Fractional Atomic Coordinates (×10^4) and Equivalent Isotropic Displacement Parameters (Å^2×10^3) for **14**. U_{eq} is defined as 1/3 of the trace of the orthogonalised U_{ij}.

**Atom**	**x**	**y**	**z**	U_{eq}
F1	7239(2)	6456(2)	5052.2(9)	23.2(4)
O1	5729(3)	6222(2)	6712.2(10)	19.6(5)
O4	3222(3)	8739(3)	6290.8(11)	21.6(5)
O5	6151(3)	2243(2)	5962.2(12)	23.1(5)
O2	8980(3)	8077(3)	5972.9(12)	22.5(5)
O3	9245(3)	7596(3)	7277.5(11)	25.7(5)
N2	3107(4)	1862(3)	5818.5(13)	19.4(6)
N6	17(4)	1520(3)	5690.6(14)	21.5(6)

Atom	x	y	z	U_{eq}
N1	4349(3)	4500(3)	5921.3(13)	17.0(6)
C3	7049(4)	8250(3)	6010.5(16)	18.9(6)
C8	2648(4)	5155(4)	5819.4(15)	18.9(6)
C2	6151(4)	6946(3)	5578.9(15)	17.8(6)
C5	4592(4)	2815(3)	5897.3(15)	18.7(7)
C4	6204(4)	7937(3)	6707.1(16)	19.6(7)
C6	1447(4)	2507(4)	5736.9(15)	18.9(6)
C1	5915(4)	5541(3)	6064.6(16)	18.9(6)
C7	1199(4)	4221(4)	5708.1(16)	19.8(7)
C12	4468(4)	8905(4)	6824.6(16)	22.1(7)
C9	7406(5)	8257(3)	7309.8(16)	21.4(7)
C10	8151(5)	9979(4)	7408.1(16)	22.2(7)
C11	10002(5)	9172(4)	7490.6(19)	26.5(7)

Table 2: Anisotropic Displacement Parameters (×10^4) for **14.** The anisotropic displacement factor exponent takes the form: $-2\pi^2[h^2a^{*2} \times U_{11}+ ... +2hka^* \times b^* \times U_{12}]$

Atom	U_{11}	U_{22}	U_{33}	U_{23}	U_{13}	U_{12}
F1	24.7(10)	20.1(8)	24.9(9)	-2.5(7)	5.6(7)	-3.8(7)
O1	23.7(11)	13.1(10)	22.0(10)	0.4(8)	2.4(9)	-4.7(9)
O4	22.4(11)	17.1(11)	25.3(11)	1.3(9)	-2.7(9)	-2.0(9)
O5	16.6(10)	14.3(10)	38.3(13)	-0.2(9)	-1.7(9)	1.8(9)
O2	14.7(10)	23.0(11)	29.8(12)	-1.0(9)	0.0(9)	-3.1(8)
O3	22.7(11)	21.2(11)	33.2(13)	-2.4(9)	-4.2(10)	3.5(9)
N2	18.1(13)	13.5(11)	26.7(14)	0.5(9)	-1.6(11)	0.0(9)
N6	19.9(13)	15.7(13)	29.0(14)	0.7(11)	-0.3(11)	-2.1(11)
N1	14.4(12)	11.6(11)	25.1(13)	0.2(9)	0.9(10)	-0.5(9)
C3	15.8(14)	14.4(14)	26.4(15)	-0.9(12)	-0.5(12)	0.4(11)
C8	19.6(15)	13.7(12)	23.5(15)	1.5(11)	0.3(12)	2.0(12)
C2	17.0(14)	14.7(14)	21.7(15)	-0.8(11)	4.8(12)	-2.0(11)
C5	18.9(14)	12.7(13)	24.4(16)	-0.6(11)	-0.7(12)	1.8(11)
C4	21.6(15)	9.4(13)	27.7(16)	-0.5(11)	-0.7(13)	-0.5(11)
C6	19.0(14)	17.1(15)	20.6(15)	0.4(12)	0.6(12)	0.0(12)
C1	16.0(15)	15.9(14)	24.7(16)	-0.5(11)	-1.0(12)	-3.0(11)
C7	16.4(14)	16.4(14)	26.5(16)	2.0(12)	1.8(12)	1.2(12)
C12	22.5(16)	20.6(15)	23.1(16)	-2.8(12)	0.1(12)	0.4(12)
C9	23.7(16)	15.8(14)	24.6(16)	0.4(12)	0.0(13)	1.9(12)
C10	22.1(15)	18.4(15)	26.1(16)	-3.3(13)	-1.3(13)	-1.3(13)
C11	23.7(17)	24.4(16)	31.4(17)	-5.3(14)	-2.9(13)	-0.6(13)

Table 3: Bond Lengths in Å for **14**.

Atom	Atom	Length/Å
F1	C2	1.382(3)
O1	C4	1.465(3)
O1	C1	1.418(4)
O4	C12	1.412(4)
O5	C5	1.245(4)
O2	C3	1.427(4)
O3	C9	1.459(4)
O3	C11	1.483(4)
N2	C5	1.356(4)
N2	C6	1.340(4)

Atom	Atom	Length/Å
N6	C6	1.335(4)
N1	C8	1.377(4)
N1	C5	1.410(4)
N1	C1	1.466(4)
C3	C2	1.533(4)
C3	C4	1.546(4)
C8	C7	1.335(4)
C2	C1	1.526(4)
C4	C12	1.524(4)
C4	C9	1.516(4)
C6	C7	1.434(4)
C9	C10	1.543(4)
C10	C11	1.524(5)

Table 4: Bond Angles in ° for **14**.

Atom	Atom	Atom	Angle/°
C1	O1	C4	111.0(2)
C9	O3	C11	90.2(2)
C6	N2	C5	120.8(2)
C8	N1	C5	120.0(3)
C8	N1	C1	120.5(2)
C5	N1	C1	119.4(3)
O2	C3	C2	109.1(2)
O2	C3	C4	115.4(3)
C2	C3	C4	102.4(2)
C7	C8	N1	121.3(3)
F1	C2	C3	112.8(2)
F1	C2	C1	109.0(2)
C1	C2	C3	103.3(2)
O5	C5	N2	121.9(3)
O5	C5	N1	119.4(3)
N2	C5	N1	118.7(3)
O1	C4	C3	105.4(2)
O1	C4	C12	108.2(3)
O1	C4	C9	107.6(2)
C12	C4	C3	112.7(2)
C9	C4	C3	116.9(3)
C9	C4	C12	105.8(2)
N2	C6	C7	121.1(3)
N6	C6	N2	118.6(3)
N6	C6	C7	120.4(3)
O1	C1	N1	109.7(2)
O1	C1	C2	106.7(2)
N1	C1	C2	114.5(3)
C8	C7	C6	117.9(3)
O4	C12	C4	111.9(2)
O3	C9	C4	116.0(2)
O3	C9	C10	91.4(2)
C4	C9	C10	118.0(3)
C11	C10	C9	85.6(2)
O3	C11	C10	91.3(2)

Table 5: Torsion Angles in ° for **14.**

Atom	Atom	Atom	Atom	Angle/°
F1	C2	C1	O1	148.7(2)
F1	C2	C1	N1	-89.8(3)
O1	C4	C12	O4	-65.8(3)
O1	C4	C9	O3	71.2(3)
O1	C4	C9	C10	178.1(3)
O2	C3	C2	F1	-27.8(3)
O2	C3	C2	C1	89.6(3)
O2	C3	C4	O1	-91.7(3)
O2	C3	C4	C12	150.6(3)
O2	C3	C4	C9	27.8(3)
O3	C9	C10	C11	-9.2(2)
N2	C6	C7	C8	-5.3(5)
N6	C6	C7	C8	173.8(3)
N1	C8	C7	C6	3.6(5)
C3	C2	C1	O1	28.6(3)
C3	C2	C1	N1	150.1(3)
C3	C4	C12	O4	50.2(3)
C3	C4	C9	O3	-47.1(3)
C3	C4	C9	C10	59.8(4)
C8	N1	C5	O5	177.0(3)
C8	N1	C5	N2	-4.5(4)
C8	N1	C1	O1	69.7(3)
C8	N1	C1	C2	-50.1(4)
C2	C3	C4	O1	26.7(3)
C2	C3	C4	C12	-91.0(3)
C2	C3	C4	C9	146.2(3)
C5	N2	C6	N6	-177.1(3)
C5	N2	C6	C7	2.1(5)
C5	N1	C8	C7	1.1(5)
C5	N1	C1	O1	-109.4(3)
C5	N1	C1	C2	130.7(3)
C4	O1	C1	N1	-136.4(2)
C4	O1	C1	C2	-11.9(3)
C4	C3	C2	F1	-150.6(2)
C4	C3	C2	C1	-33.2(3)
C4	C9	C10	C11	-129.8(3)
C6	N2	C5	O5	-178.7(3)
C6	N2	C5	N1	2.7(4)
C1	O1	C4	C3	-9.6(3)
C1	O1	C4	C12	111.1(3)
C1	O1	C4	C9	-135.1(3)
C1	N1	C8	C7	-178.0(3)
C1	N1	C5	O5	-3.9(4)
C1	N1	C5	N2	174.7(3)
C12	C4	C9	O3	-173.4(2)
C12	C4	C9	C10	-66.5(3)
C9	O3	C11	C10	-9.6(2)
C9	C4	C12	O4	179.1(2)
C9	C10	C11	O3	9.1(2)

Atom	Atom	Atom	Atom	Angle/°
C11	O3	C9	C4	131.8(3)
C11	O3	C9	C10	9.4(2)

Table 6: Hydrogen Fractional Atomic Coordinates (×10^4) and Equivalent Isotropic Displacement Parameters (Å^2×10^3) for **14.** U_{eq} is defined as 1/3 of the trace of the orthogonalised U_{ij}.

Atom	x	y	z	U_{eq}
H4	3229.14	9561.08	6063.12	32
H2	9460.59	7821.02	6415.34	34
H3	6692.04	9320.38	5849.73	23
H8	2506.17	6268.4	5828.28	23
H2A	4963.83	7314.86	5414.61	21
H1	7023.34	4882.71	6055.29	23
H7	64.68	4666.83	5614.87	24
H12A	3896.91	8544.15	7235.91	26
H12B	4779.02	10034.77	6877.6	26
H9	6796.54	7890.23	7718.88	26
H10A	8049.97	10664.31	7016.47	27
H10B	7707.75	10518.37	7806.97	27
H11A	10923.27	9589.33	7188.78	32
H11B	10441.42	9174.4	7948.55	32
H6A	-1140(30)	2010(50)	5720(20)	46(9)
H6B	90(70)	397(14)	5750(20)	46(9)

.
